# Failed resolution as a mechanistic basis of difficult-to-treat rheumatoid arthritis: stromal–myeloid crosstalk in the synovium and emerging resolution-restoring therapies

**DOI:** 10.3389/fimmu.2026.1935792

**Published:** 2026-09-07

**Authors:** Wenliang Wei, Jianzhong Xu

**Affiliations:** Department of Orthopedics, The First Affiliated Hospital of Zhengzhou University, Zhengzhou, Henan, China

**Keywords:** difficult-to-treat rheumatoid arthritis, inflammation resolution, rheumatoid arthritis, stromal–myeloid crosstalk, synovial fibroblast

## Abstract

Modern disease-modifying therapy has transformed rheumatoid arthritis. Even so, many patients cycle through biological and targeted synthetic drugs of distinct mechanisms without achieving sustained remission. This is the difficult-to-treat phenotype defined by the European Alliance of Associations for Rheumatology. We argue that in a large fraction, refractory disease is not simply inflammation that is too strong or too drug-resistant. It is better understood as an active failure of the program meant to end inflammation. Single-cell and spatial dissection of the synovium lets us recast the rheumatoid joint as a niche of failed resolution. In this niche, imprinted pathological fibroblasts and a macrophage compartment stripped of its pro-resolving, efferocytic subsets both work against resolution. Their reciprocal, largely feed-forward signaling, we propose, assembles a self-sustaining stromal–myeloid circuit: a “failed-resolution switch”. Lipid-mediator, metabolic, and epigenetic determinants reinforce it, and synovial microarchitecture stabilizes it. A switch built from redundant, mutually reinforcing arms would explain the therapeutic ceiling in refractory disease. Blocking any single mediator can lower inflammatory output while leaving resolution unrestored. Because the framework describes failed resolution rather than every route to refractoriness, it applies most directly to the persistent-inflammatory subset of difficult-to-treat disease. For that subset, it defines a measurable mechanistic state that can be stratified and which is open to intervention. It also motivates resolution-restoring strategies: pro-resolving mediators, macrophage reprogramming, and fibroblast- and crosstalk-directed interventions. Early clinical evidence, from the instructive failure of the granulocyte-macrophage colony-stimulating factor (GM-CSF) blockade to a proof-of-concept signal for programmed cell death protein 1 (PD-1) agonism, points toward reversing the disease rather than only suppressing it.

## 1 Introduction

Rheumatoid arthritis (RA) is the most common inflammatory arthritis and a leading cause of chronic pain, disability, and shortened life expectancy worldwide (1). Three advances over three decades transformed the outlook for many patients. These are a deeper grasp of its immunopathogenesis, a treat-to-target strategy, and a growing set of conventional, biologic, and targeted synthetic disease-modifying antirheumatic drugs (DMARDs) (2, 3). RA is driven by amplified pro-inflammatory signaling, centered on tumor necrosis factor (TNF), interleukin-6 (IL-6), the Janus kinase pathway, and granulocyte-macrophage colony-stimulating factor (GM-CSF) (4). This signaling acts against a background of lost self-tolerance marked by anti-citrullinated protein antibodies (ACPA). ACPA recognize citrullinated epitopes and appear years before clinical disease (5–9). Blocking these pathways, starting with anti-TNF biologics, confirmed that damping a dominant inflammatory circuit can control disease (10, 11).

Roughly 40% of patients respond inadequately to any single biologic, and a subset remains refractory after cycling through agents with distinct mechanisms of action (12). These are the patients now captured formally by the European Alliance of Associations for Rheumatology (EULAR) definition of difficult-to-treat RA (D2T-RA) (13). Refractoriness is not one entity. It ranges from persistent inflammatory disease that resists successive targeted therapies to disease activity largely uncoupled from measurable inflammation (14). The R4RA trial, which used synovial biopsy-driven stratification, fits this picture. Resistance to all biologics tested tracked with a stromal or fibroblast molecular signature rather than a lymphoid one (12). Existing medications are not entirely suppressive, and several influence pro-resolving mechanisms, as detailed below. None were designed to restore resolution, and no trial has measured whether they did. Lowering the intensity of inflammation is not the same as ending its persistence. Some patients seem locked in a chronic state that more immunosuppression does not release.

This deadlock may be explained by a complementary framework. Acute inflammation is not switched off passively. It ends through an active, genetically programmed process. That process has three coordinated arms: specialized pro-resolving mediators (SPMs), efferocytic clearance of apoptotic cells, and a switch of macrophages from a pro-inflammatory to a pro-resolving phenotype (15, 16). Where this program fails, inflammation does not resolve but becomes chronic, a “resolution deficit” now implicated across chronic inflammatory disease (17). Single-cell and spatial atlases redefine the synovium as an ecosystem of specialized stromal and myeloid states, among them pathogenic fibroblast subsets that sustain inflammation and drive tissue damage (18–20). Resident macrophages that form a protective, homeostatic barrier are displaced in active disease, while pro-resolving macrophage populations associated with remission are depleted or dysfunctional (21, 22). Together these findings invite a reframing of persistent and difficult-to-treat RA as inflammation that fails to resolve, not merely inflammation that is too strong.

We build that reframing into an integrated mechanistic account. Prior reviews organize around cytokine amplification or around a single cell lineage. We instead take failed resolution as the unifying lens and place bidirectional fibroblast–myeloid crosstalk at its center. Our proposal is that reciprocal stromal–myeloid signaling forms a self-sustaining “non-resolution switch”, able to lock the synovium in a chronic state (23). We then map the clinical phenotype of D2T-RA, most directly its persistent-inflammatory form, onto this circuitry. Alongside it we survey the emerging resolution-restoring strategies: SPM-based approaches, macrophage reprogramming, and fibroblast-directed and crosstalk-node therapies. We set these against their clinical trial evidence, including instructive negative results such as GM-CSF blockade in advanced disease (24).

## 2 The resolution paradigm: how acute inflammation is meant to end

Resolution of acute inflammation is now read as an actively orchestrated, genetically encoded program with its own effectors and endpoints, not the passive fading of pro-inflammatory signals (15, 25). In a self-limiting response, the tissue that mounted inflammation later switches its biochemical output to shut it down: neutrophil recruitment ceases, apoptotic cells are cleared, and tissue architecture is restored. That active view recasts chronic inflammation. Persistence need not signal an ongoing pro-inflammatory drive alone. It may equally signal failure of the program that should have closed the response (17). Because resolution effectors differ from the initiators of inflammation, they form a separate control axis that can fail after the initiating stimulus has passed.

The program involves three coordinated steps: a temporally staged lipid-mediator class switch, efferocytic clearance of apoptotic cells, and reprogramming of macrophages to a pro-resolving state. As acute inflammation peaks, prostaglandin E2 induces a switch in lipoxygenase expression. This steers arachidonic acid metabolism away from pro-inflammatory leukotrienes and toward lipoxin A4, a signal that tells leukocytes to “stop” recruiting (16). Similar class switching yields the specialized pro-resolving mediators (SPMs) derived from omega-3 fatty acids: the resolvins, the protectins, and the macrophage-derived maresins (26). These mediators actively promote the resolution phase rather than merely blunting inflammation (27, 28). Stable lipoxin analogs make the causal role of these lipids explicit by limiting neutrophil trafficking *in vivo* (29), and resolvin E1 signals macrophages to phagocytose apoptotic neutrophils (30). Efferocytosis itself drives resolution rather than passively disposing of debris. Metabolites of apoptotic cell material sustain a continual efferocytosis loop that clears the inflammatory field and reprograms the engulfing macrophage (31). Through these signals, the macrophage converts to a pro-resolving, reparative phenotype that supports tissue regeneration (32).

Failure at any step blocks closure, and self-limiting responses become non-resolving, chronic ones. A class switch that does not fire, ineffective efferocytosis, or a macrophage community that never converts to a pro-resolving phenotype each leaves the tissue exposed long after the initiating insult. This “resolution deficit” is increasingly recognized as a common basis of chronic inflammatory disease, ranging from arthritis to atherosclerotic cardiovascular disease (17, 33). Viewed this way, SPMs serve as endogenous immunoresolvents that hasten the return to homeostasis without the widespread immunosuppression of traditional anti-inflammatory agents (27). Resolution pharmacology thereby becomes a therapeutic principle in its own right (34, 35). The question that follows is not why rheumatoid arthritis inflammation is so robust, but where its resolution fails. That failure plays out in the synovium.

## 3 The rheumatoid synovium as a niche of failed resolution

In rheumatoid arthritis, the synovium changes from a thin, homeostatic lining into a hyperplastic, immune-infiltrated tissue. It behaves less like a site of ongoing injury than like one where the resolution program has failed. The thin healthy lining, only one to three cells deep, plays a nutritive and barrier role. In established disease, it expands into an aggressive, locally invasive pannus that erodes cartilage and bone (1, 36). Seen through the resolution paradigm, the striking feature of this tissue is its permanence more than the sheer intensity of the infiltrate. The coordinated shutdown that normally terminates an acute response never arrives, and the synovium stays in a chronic, self-perpetuating state. Below we treat the synovium as the niche where failed resolution is enacted and ask where the program breaks down.

### 3.1 From a self-limiting response to a locked synovium

Self-limiting arthritis shows what the rheumatoid joint fails to do. Acute and reactive arthritides mount a vigorous synovial response that nonetheless resolves, clearing infiltrating leukocytes and restoring the lining. The rheumatoid synovium, by contrast, becomes locked in a state that never closes (36). Several features mark that transition. Ectopic lymphoid structures organize within the inflamed synovium and locally sustain production of class-switched, citrullinated-protein-specific autoantibodies, embedding a self-renewing adaptive stimulus in the tissue instead of letting the response subside (37). Neutrophil extracellular traps add a continual source of citrullinated autoantigen that further fuels the autoantibody response (38). The structural consequences themselves signal non-resolution. Bone erosion continues at the pannus–bone interface through sustained osteoclast activity and is associated with the failure to return the joint to homeostasis rather than with any single acute insult (39). Within the resolution framework these are not simply markers of severity. They are evidence that the endpoint of an inflammatory response, cessation and repair, is never reached.

### 3.2 Single-cell and spatial atlases localize where resolution fails

**Figure 1:**
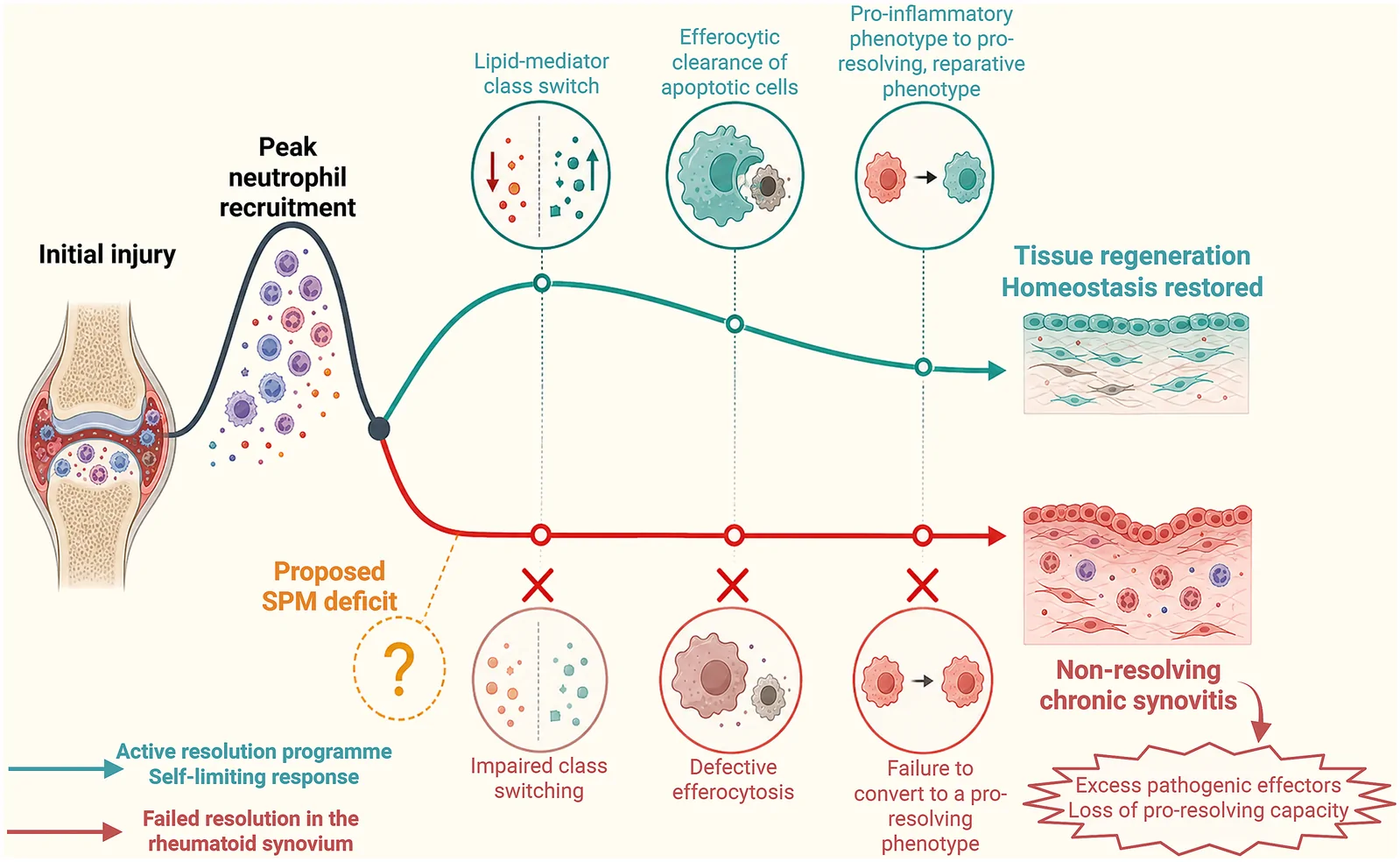
Resolution as an active program and its failure in the rheumatoid synovium. Schematic model (illustrative, not to scale; not quantitative data) contrasting the fate of a shared acute inflammatory stimulus along two parallel timelines of inflammatory intensity. In physiological resolution (upper track), the response runs through an ordered program: neutrophil recruitment peaks and ceases; a lipid-mediator class switch redirects arachidonic-acid metabolism from prostaglandin E2 and leukotriene B4 toward lipoxins and ω-3-derived specialized pro-resolving mediators (SPMs); efferocytosis clears apoptotic cells; and macrophages switch to a pro-resolving, reparative phenotype, returning the tissue to homeostasis as the curve resolves to baseline. In the rheumatoid synovium (lower track), the same stimulus meets a program that breaks at three definable nodes (✗): a failed class switch with a deficit of SPMs, defective efferocytosis, and an arrested macrophage phenotype switch. With these effector steps failing, the inflammatory curve does not return to baseline and the tissue enters a persistent, non-resolving state. The three shared nodes establish the coordinate system used in the mechanistic sections that follow. LTB4, leukotriene B4; PGE2, prostaglandin E2; SPM, specialized pro-resolving mediator. Figure created with BioRender.com.

High-resolution profiling has redefined the synovium as an ecosystem of specialized stromal and myeloid states and has begun to pinpoint where resolution fails inside it. The first Accelerating Medicines Partnership atlas combined single-cell transcriptomics with mass cytometry to resolve discrete inflammatory cell states. It identified an expansion of THY1^+^ HLA-DRA^hi^ sublining fibroblasts and pro-inflammatory IL1B^+^ monocytes that distinguish active disease (19). A second-generation atlas of more than 300,000 cells then deconstructed the synovium into six recurrent cell-type abundance phenotypes whose composition tracks with, and may predict, therapeutic response. The tissue is therefore a set of definable ecological configurations, not a uniform inflammatory mass (20). Two observations locate the resolution defect on this cellular map. The resident macrophage barrier that normally shields the synovial lining is disrupted in active disease and replaced by monocyte-derived cells (21). Macrophage subsets related to clinical remission are depleted or functionally impaired in persistent disease (22). The synovium therefore carries an excess of pathogenic effectors and a shortfall of the cells that would drive the tissue back toward homeostasis (**Figure 1**). Both compartments are examined in detail below.

Characterized as a niche of failed resolution, the synovium raises one question: which cell actors and signaling circuits hold it in that state? Imprinted fibroblast subsets supply a persistent inflammatory scaffold. The macrophage compartment cannot complete the switch toward resolution. Reciprocal signaling couples the two. Whether single-cell atlases capture reversible or fixed ecological configurations bears directly on whether resolution can be therapeutically restored. The next section presents a mechanistic account of the fibroblast subsets that contribute to this self-sustaining failure.

## 4 Synovial fibroblast heterogeneity drives persistent inflammation

In the past, synovial fibroblasts were thought of as just passive structural cells. Now they are considered a heterogeneous population of imprinted effector cells that drive and maintain rheumatoid inflammation. In rheumatoid arthritis, fibroblast-like synoviocytes (FLS) do not merely respond transiently to cytokines derived from leukocytes. They exhibit stable, aggressive characteristics and divide into anatomically distinct subgroups with different and often opposing functions (18). The stromal compartment supplies both the inflammatory milieu and the machinery of tissue destruction, and it does so well beyond the life of the immediate stimulus (23, 40). Which subsets exist, what they do, and why their pathogenic behavior persists is therefore central to why the rheumatoid synovium never resolves.

### 4.1 Subset identity and effector functions

At single-cell resolution, a fibroblast’s position in the synovium predicts its function, with lining and sublining subsets taking largely opposing roles (**Table 1**). Sublining fibroblasts marked by FAPα and THY1 (CD90) act as immune-effector cells that amplify inflammation. FAPα^+^THY1^-^ fibroblasts, by contrast, sit in the lining and mediate the invasive, bone- and cartilage-destructive behavior of the pannus. Selective expansion of each subset in experimental arthritis confirmed the division of labor (18). Further markers, including podoplanin, cadherin-11, and CD34, stratify these populations and delineate a THY1^+^CD34^-^ perivascular subset associated with synovitis (41). An endothelium-derived NOTCH3 gradient drives the differentiation and positional identity of sublining fibroblasts, so the pathogenic architecture is actively specified rather than passively acquired (42). Cadherin-11 governs formation and condensation of the synovial lining and licenses its invasive phenotype (43, 44). RANKL and matrix-degrading enzymes released by activated FLS act directly on bone erosion. Their capacity to spread through blood vessels and seed disease in previously unaffected joints indicates that they behave more like transformed cells than merely reactive ones (45).

**Table 1:** Key fibroblast and macrophage subsets in the rheumatoid synovium: markers, anatomical niche, and role in failed resolution.

Compartment	Subset	Key markers	Anatomical niche	Principal function	Role in resolution	Behavior in active/D2T RA	Ref.
Stroma	Sublining immune-effector FLS	FAPα^+^ THY1^+^ (CD90)	Sublining	Amplify inflammation; immune effector function	Anti-resolution	Expanded	(18)
Stroma	Lining-destructive FLS	FAPα^+^ THY1^-^	Lining layer	Invasion; bone/cartilage destruction	Anti-resolution	Expanded	(18)
Stroma	Perivascular FLS	THY1^+^ CD34^-^ (NOTCH3-specified)	Perivascular	Accompany synovitis; niche construction	Anti-resolution	Expanded/niche-localized	(41, 42)
Stroma	Imprinted FLS state	DNA-methylation signature/HOX positional memory/glycolytic shift	—	Inflammatory memory; self-sustained activation	Anti-resolution (memory)	Stably maintained	(46, 48, 49)
Myeloid	Resident barrier macrophage	CX3CR1^+^ TREM2^+^ (tight junctions)	Lining barrier layer	Physical/functional barrier; restrain inflammation	Barrier/homeostatic	Disrupted; replaced	(21)
Myeloid	Pro-resolving macrophage	MerTK^+^ TREM2^hi^/MerTK^+^ LYVE1^+^	Lining/tissue	Efferocytosis; SPM production	Pro-resolution (negative-feedback brake)	Depleted/dysfunctional	(22)
Myeloid	Monocyte-derived inflammatory macrophage	TREM2^-^ (CX3CR1-driven differentiation)	Sublining	Amplify inflammation; replace barrier	Anti-resolution	Replacement/expansion	(21, 59)

### 4.2 Epigenetic and metabolic imprinting: an inflammatory memory

Stable epigenetic and metabolic imprinting gives rheumatoid FLS an inflammatory memory. Genome-wide analysis has identified a rheumatoid-specific DNA methylation signature in synovial fibroblasts. The signature survives repeated passages in culture and matches their aggressive, pathogenic phenotype. The disease state is therefore intrinsic to the cell, not externally imposed (46). Interleukin-1 alters methylation at specific loci through DNA methyltransferase activity (47). This is a crucial molecular route from transient inflammation to stable transcriptional change. Epigenetic patterns also set anatomical identity: HOX gene programs govern joint-specific fibroblast behavior and differential responsiveness to tumor necrosis factor. This helps explain why disease is expressed differently across joints, and why fibroblasts retain location-specific function when removed from their tissue (48). Rheumatoid FLS reprogram toward glycolysis in a Warburg-like manner. Blocking glycolytic flux lowers both their inflammatory output and their invasiveness, tying the imprinted phenotype to a targetable bioenergetic state (49). Together, these programs let fibroblasts hold an activated phenotype independently of the immediate cytokine environment, uncoupled from any ongoing acute stimulus.

### 4.3 A second track: TGF-β signaling and the fibroid pathotype

Not every form of non-resolution is inflammatory. A second track runs through fibrosis, and transforming growth factor beta (TGF-β) organizes it. The pathway is constitutively upregulated in rheumatoid synovial fibroblasts compared with osteoarthritic ones, although that study read out matrix turnover and not myofibroblast conversion (50). As established in other fibrotic tissues, TGF-β converts fibroblasts into myofibroblasts, sustains matrix deposition, and can leave activation self-perpetuating once mechanical and transcriptional feedback engages (51, 52). Adenoviral overexpression of TGF-β1 in the rat knee alone produced dense arthrofibrosis, α-SMA^+^ myofibroblasts derived from resident fibroblasts, and cartilage metaplasia (53). That model is supraphysiological and not rheumatoid, so it shows what the pathway can do, not that it does so in patients.

In paired synovial biopsies taken before treatment and 6 months later, fibroblast TGF-β signaling was spatially patterned, not uniform. Patients who did not reach remission already showed raised pro-fibrotic signaling in the perivascular niche at baseline, with fibroblasts expressing high levels of COMP. After treatment, the biopsies showed marked depletion of the immune compartment alongside expansion of pro-fibrotic niches. Endothelial Notch signaling sat upstream, inducing TGF-β isoforms while repressing TGF-β receptors, setting a sensitivity gradient outwards from the vessel. Inhibiting Notch and TGF-β reversed that expansion in patient-derived organoids (54). The post-treatment finding applies to the treated biopsies as a group and was not stratified by remission status.

The upstream regulator is the same endothelial Notch axis that patterns sublining fibroblast identity (42), so the fibrotic track is continuous with the architecture already set out. Treatment suppressed the immune compartment while the stromal track advanced, which is a second and independent instance of suppression without resolution. At tissue level, the fibroblastic pauci-immune pathotype defined in early disease may mark this track (55, 56).

This is preliminary evidence, not conclusive proof. Only one human study of the joint has been published, in 2026, and the pathotype link rests on small numbers and one drug class. Treated as a hypothesis, the fibrotic pathway complements the framework developed here. The inflammatory circuit holds the synovium open, and fibrosis lays down a track that can persist once that circuit is damped.

Synovial fibroblasts are therefore a node at which the brake on inflammation fails. If the pathogenic fibroblast phenotype is indeed epigenetically and metabolically imprinted, then cytokine suppression will not suffice to return the stromal compartment to rest. Three approaches follow: subset-selective targeting that spares protective lining or sublining populations, reversal of the pathogenic epigenetic imprint, and interference with the metabolic programs that maintain it. This approach is captured by the idea of restoring synovial homeostasis rather than only suppressing its inflammatory products (23).

Real uncertainties temper the ambition: whether the imprinted phenotype is truly reversible *in vivo* and whether subsets can be hit specifically enough to spare normal joint maintenance (49). The fibrotic track adds a further caveat, since damping the inflammatory circuit may leave the pro-fibrotic one running. The fibroblast clearly supplies a persistent, self-renewing scaffold for inflammation. How that scaffold is sustained through reciprocal signaling depends on the myeloid compartment.

## 5 Synovial macrophages and impaired resolution

Synovial macrophages sit at the center of resolution in the joint. Their failure to complete the switch from a pro-inflammatory to a pro-resolving state is a defining feature of persistent rheumatoid arthritis. In health, resident macrophages maintain tissue homeostasis and actively contain inflammation. In disease, the composition and function of the compartment shift so that the resolution program is left unexecuted (21). Macrophages both execute efferocytosis and mark the endpoint of an inflammatory response by their phenotypic conversion. Their behavior therefore weighs heavily on whether the synovium can return to homeostasis or stays locked in chronic activity (22).

### 5.1 Barrier disruption and monocyte-derived replacement

The uninjured synovium is protected by locally renewing resident macrophages that form a physical barrier. Tissue-resident macrophages generally sustain themselves locally through adult life, with limited input from circulating monocytes (57, 58). The population is therefore set by the tissue, not continuously imported from the bloodstream. Within the joint, a CX3CR1^+^TREM2^+^ subset forms an epithelial-like layer, tightly bound by tight junctions, that shields the synovium physically and functionally. This internal barrier prevents entry and activation of inflammatory cells (21). Rheumatoid arthritis damages that barrier. The network of tight junctions collapses, allowing entry of TREM2^-^, monocyte-derived macrophages with a pro-inflammatory phenotype (21). A structure that normally inhibits inflammation is thereby converted into one that amplifies it. The replacement monocytes are themselves biased toward an inflammatory fate, with CX3CR1 signaling supporting their maturation into pro-inflammatory macrophages in other tissues (59). The consequence is an ecological substitution: a self-renewing homeostatic compartment is replaced by a continuously replenishing inflammatory one, and a cellular brake on the response is removed.

### 5.2 Defective efferocytosis and depletion of pro-resolving subsets

Two functional defects sit on top of this substitution and hinder the macrophage compartment from driving resolution: impaired efferocytic clearance of apoptotic cells and failure of the pro-resolving phenotypic switch. In the rheumatoid synovium, the clearance loop is interrupted, so uncleared apoptotic cells undergo secondary necrosis and add to the inflammatory load (31). MER tyrosine kinase (MerTK) mediates efferocytosis and links it to tissue repair, and its loss delays resolution in a myocardial infarction model (60). Annexin A1 released from apoptotic cells acts through formyl peptide receptors to dampen inflammatory activation and upregulate suppressor-of-cytokine signaling (61). The decisive point in rheumatoid arthritis is that the populations equipped for these functions are themselves depleted or dysfunctional in persistent disease. MerTK^+^TREM2^hi^ and MerTK^+^LYVE1^+^ subsets are associated with clinical remission and carry a pro-resolving, lipid-mediator–producing signature. Both are diminished in active and difficult-to-treat disease, where inflammatory states dominate the compartment instead (**Table 1**) (22). The macrophage switch that should terminate inflammation is thus impaired both structurally and functionally.

A stalled switch makes the myeloid compartment a second node of failed resolution and points to a distinct therapeutic axis. If persistent disease reflects a shortage of pro-resolving, efferocytic macrophages as much as an excess of inflammatory ones, restoring resolution will mean replenishing or reprogramming the compartment. That means enhancing efferocytosis, engaging the MerTK axis, or inducing the pro-resolving phenotype, rather than suppressing inflammatory mediators alone. One question is whether loss of barrier and pro-resolving macrophages is a primary event or a downstream effect of the stromal environment. Another is whether recruited inflammatory macrophages can be reprogrammed *in situ* instead of merely depleted (21). The depletion of pro-resolving populations and the expansion of inflammatory ones appear to be held in place by continuous signaling exchange with the fibroblast compartment. We argue that this reciprocal stromal–myeloid dialogue, rather than either cell type by itself, locks the synovium in a non-resolving state.

## 6 Stromal–myeloid crosstalk circuits that lock non-resolution

Failed resolution in the rheumatoid synovium is not best read as a defect intrinsic to fibroblasts or to macrophages alone. It is better read as an emergent property of the signaling that couples them. Pathological synovial fibroblasts and dysregulated myeloid populations each carry cell-autonomous features opposing resolution. Neither, acting alone, easily accounts for the stability with which the tissue stays non-resolving. That stability, we propose, is a circuit property. Stromal and myeloid compartments instruct one another continuously through paired, largely feed-forward signaling arms, so the activated phenotype of each cell is sustained by the output of the other. On this reading, the immune–fibroblast–bone interactions described in RA (62) become less a list of parallel pathways than the wiring diagram of a self-reinforcing loop. The two directional arms are taken apart below, with the ligand-receptor axes that carry each and the level of evidence behind them (**Table 2**).

**Table 2:** Ligand–receptor axes of the stromal–myeloid circuit in rheumatoid synovium.

Ligand (source cell)	Receptor (target cell)	Cellular and molecular consequence	Level of evidence	Ref.
GM-CSF (synovial fibroblast)	GM-CSF receptor α/βc (infiltrating monocyte)	Supports monocyte survival and inflammatory differentiation; converts a transient influx into a durable effector pool	Fibroblast–monocyte coculture	(63)
CXCL10, CCL19 (chemokine-high fibroblast states)	CXCR3, CCR7 (myeloid and lymphoid cells)	Establishes sublining chemokine gradients; retains myeloid cells where fibroblast cues are strongest; supports ectopic lymphoid organization	Tissue expression and spatial association	(18, 64)
Notch ligands (endothelium)	NOTCH3 (perivascular fibroblast)	Specifies perivascular fibroblast identity along a sublining gradient; anchors the myeloid compartment to the vascular niche	Coculture and murine perturbation	(42)
TNF-α, IL-1β (macrophage)	TNFR1, IL-1R (fibroblast)	Re-activates fibroblasts and reinforces fibroblast GM-CSF output; IL-1 additionally imposes a DNMT-dependent methylation imprint	Fibroblast stimulation studies	(47)
IL-6 with soluble IL-6R (macrophage)	gp130 (fibroblast, trans-signaling)	Shifts fibroblast output toward pro-inflammatory rather than regenerative programs	Receptor biology and cytokine studies	(65)
HBEGF (inflammatory macrophage)	EGFR (fibroblast)	Induces fibroblast invasiveness	Coculture, pharmacological EGFR blockade and synovial explant	(66)
RANKL (fibroblast, T cell)	RANK (osteoclast precursor)	Drives osteoclastogenesis and bone erosion; an output of the circuit rather than a driver of its persistence	Tissue expression and murine models	(67, 68)
Gas6, protein S (macrophage, resolution arm)	MerTK (macrophage)	Couples efferocytosis to a resolution-phase phenotype; the arm depleted in persistent disease and induced incidentally by TNF blockade	Non-joint injury models; ex vivo RA macrophages	(60, 90)
Annexin A1 (apoptotic cell)	FPR2/ALX (monocyte, macrophage)	Dampens inflammatory activation and upregulates suppressor-of-cytokine signaling	Cell and murine studies	(61)

### 6.1 The stroma-to-myeloid arm

The first arm runs from stroma to myeloid cells. Pathological fibroblasts recruit, retain, and reprogram infiltrating monocytes toward a persistently pro-inflammatory phenotype, instead of allowing their clearance or their maturation into barrier-type macrophages.

Survival is the first output. IL-1β and TNF-α act on synovial fibroblasts to induce fibroblast-derived GM-CSF (63). This engages GM-CSF receptor α and the common β chain on infiltrating monocytes and helps keep them alive. A transient influx is thereby converted into a durable effector pool.

Retention follows survival. CXCL10^+^CCL19^+^ and other chemokine-high fibroblast states expand in inflamed synovium. The gradients they establish engage CXCR3 and CCR7 on myeloid and lymphoid cells, concentrating them in the sublining (18, 64). Position matters: it places these cells where fibroblast-derived cues are strongest.

Endothelium-derived Notch ligands signal through NOTCH3 on perivascular fibroblasts and organize their identity along a sublining gradient (42). The same vascular niche is where recruited monocytes gather, so the fibroblast-built architecture also anchors the myeloid compartment. The net effect is displacement of the self-renewing barrier macrophages that normally enforce resolution by monocyte-derived cells held in an activated state (21). This is a stromally instructed shift toward a non-resolving myeloid landscape.

### 6.2 The myeloid-to-stroma arm

The second arm closes the loop, turning fibroblast-instructed macrophages from downstream effectors into upstream drivers. Macrophage-derived TNF-α, IL-1, and IL-6 are the principal return signals. Acting through TNFR1 and IL-1R, they re-activate fibroblasts and reinforce the fibroblast programs that generated the first arm, GM-CSF production among them. The two arms are therefore mutually self-sustaining rather than sequential.

IL-1 alters DNA methylation at defined loci in synovial fibroblasts through DNMT-dependent mechanisms (47), the molecular basis for that inflammatory memory. The loop therefore leaves a heritable mark and not merely a transient one. Its IL-6 component is thought to amplify the circuit selectively: the soluble IL-6 receptor allows trans-signaling through gp130 on fibroblasts, which favors pro-inflammatory over regenerative outputs (65).

One myeloid-to-stromal axis has been tested directly. Inflammatory macrophages expressing HBEGF act on fibroblast EGFR to induce an invasive fibroblast phenotype, shown by coculture, by pharmacological EGFR blockade, and in synovial explants (66). It is the clearest experimental demonstration that a myeloid signal can install a pathogenic stromal program.

The loop also couples to bone. Cytokine-controlled RANKL expression by synovial fibroblasts (67), with RANK-independent osteoclast formation driven by TNF and IL-6 (68), channels the same crosstalk into structural erosion. A broader cytokine- and ACPA-driven osteoclast program reinforces the coupling, with autoantibodies against citrullinated and glycosylated targets promoting osteoclastogenesis and bone loss (39, 69–71). TNF meanwhile restrains repair through Dickkopf-1-mediated suppression of bone formation (72). Each return signal thus perpetuates the stromal activation state and translates it into irreversible damage.

### 6.3 An integrated “failed-resolution switch”

**Figure 2:**
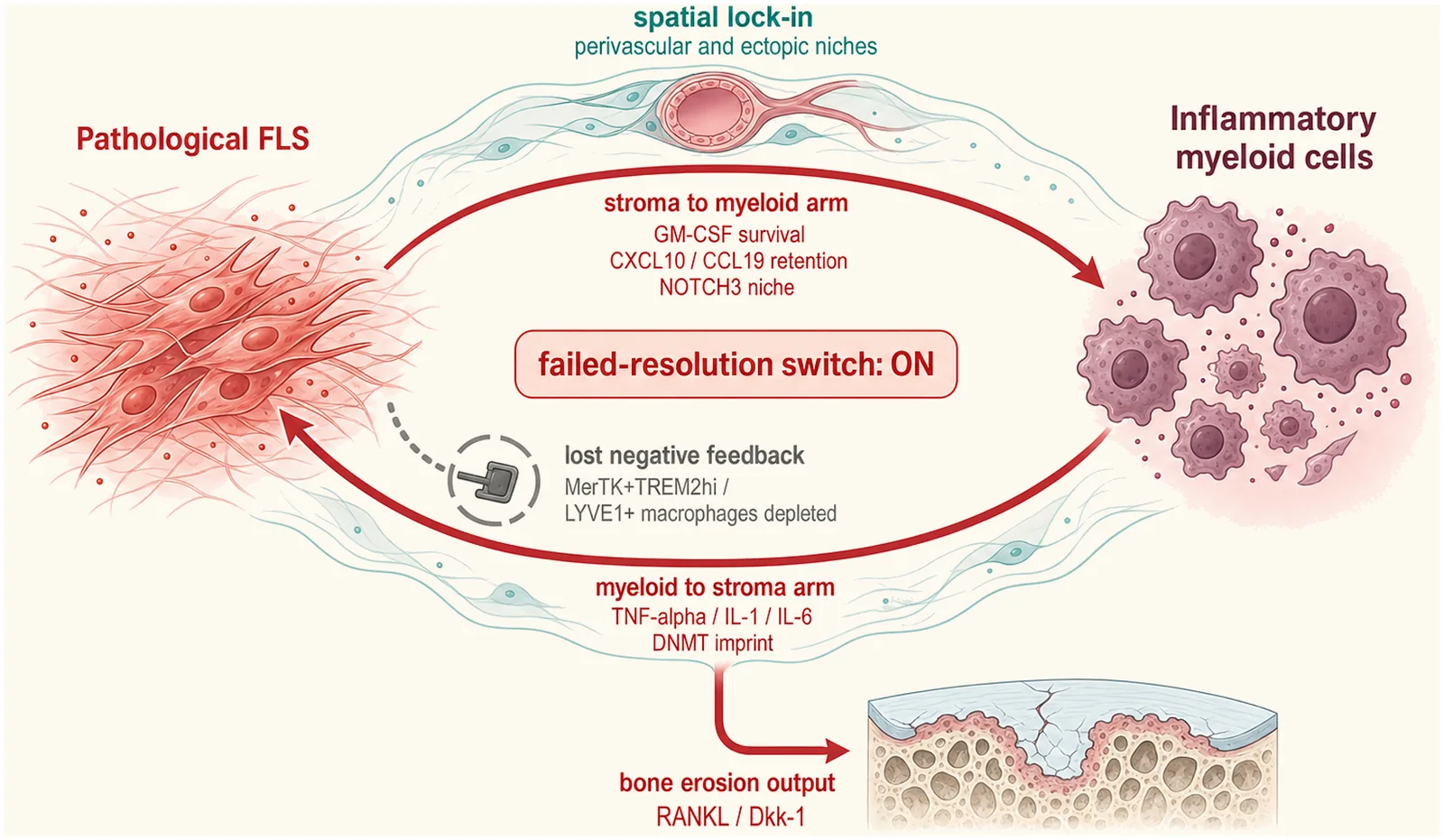
A self-sustaining stromal–myeloid circuit locks the synovium in a failed-resolution state (the “failed-resolution switch”). Proposed integrative model in which reciprocal, largely feed-forward signaling between pathological fibroblast-like synoviocytes (stroma, left) and monocyte-derived pro-inflammatory macrophages (myeloid, right) forms a closed, self-reinforcing loop. In the stroma→myeloid arm (upper arc), fibroblast-derived GM-CSF induced by IL-1β and TNF sustains monocyte viability; CXCL10/CCL19 gradients retain myeloid cells in the sublining; and an endothelium-derived NOTCH3 perivascular niche anchors their accumulation, together displacing the CX3CR1^+^TREM2^+^ barrier macrophages that normally enforce resolution. In the myeloid→stroma arm (lower arc), macrophage-derived TNF-α, IL-1, and IL-6 (the last amplified through trans-signaling via the soluble IL-6 receptor) re-activate fibroblasts and reinforce their GM-CSF program, while IL-1-driven, DNMT-dependent DNA-methylation changes leave a heritable epigenetic mark (inflammatory memory). Two annotations mark the shift from a transient to a locked circuit: loss of negative feedback through depletion or dysfunction of MerTK^+^TREM2^hi^ and MerTK^+^LYVE1^+^ pro-resolving macrophages (the missing brake), and spatial locking by ectopic lymphoid structures and the perivascular niche. The switch is held “on” by redundant, mutually reinforcing arms rather than any single mediator, so blockade of one mediator leaves the reciprocal arm able to rebuild the loop. A bone-erosion branch (RANKL-dependent and RANK-independent osteoclastogenesis; TNF–Dkk-1 suppression of bone formation) is depicted as an output of the circuit, not a driver. The model is a testable framework; much of the supporting evidence is associative, from cross-sectional single-cell and spatial data. CCL19, C-C motif chemokine ligand 19; CXCL10, C-X-C motif chemokine ligand 10; Dkk-1, Dickkopf-1; DNMT, DNA methyltransferase; GM-CSF, granulocyte–macrophage colony-stimulating factor; IL, interleukin; RANK(L), receptor activator of nuclear factor-κB (ligand); TNF, tumor necrosis factor. Figure created with BioRender.com.

Reassembled, the arms describe a switch rather than a gradient. We propose that the paired feed-forward loops of the stromal–myeloid circuit functionally replace the negative-feedback architecture on which physiological resolution depends. That replacement, rather than the magnitude of any single mediator, is what we suggest defines the failed-resolution state (**Figure 2**).

Two features would turn such a circuit from transient into locked. The first is loss of the counterweight from within. In chronic disease, the naturally occurring brake that would otherwise close the loop is absent because the pro-resolving macrophage subsets are depleted or dysfunctional (22). The second is stabilization in space. Ectopic lymphoid structures organized within the sublining (37) and the perivascular fibroblast niche create durable microanatomical compartments that concentrate and shield the reciprocal signals. Tissue architecture may thus hold the switch on as much as soluble mediators do.

Suppose a lack of resolution is sustained by redundant, mutually strengthening arms rather than by one dominant node. Blocking a single mediator would then leave the reciprocal arm intact to rebuild the loop. That gives a circuit-level account of why single-target inhibition can suppress inflammatory output without restoring resolution. It also fits the finding that patients refractory across multiple targeted therapies are enriched for a stromal and fibroblast-dominated synovial signature (12). The therapeutic problem is thereby recast, away from intensifying suppression of individual mediators and toward interrupting the circuit and re-arming its lost negative feedback (23).

Much of the supporting evidence is associative, and most axes in **Table 2** rest on expression or co-expression rather than on perturbation in human tissue. The failed-resolution switch is therefore best treated as a testable model, one whose causal structure and reversibility await prospective and interventional study. Three questions frame that agenda. Which node, if any, is rate-limiting for loop re-entry after therapy? Can the imprinted fibroblast state be reversed, or only contained? And is restoring the depleted pro-resolving macrophage compartment sufficient to break the switch, or must it be paired with stromal reprogramming?

## 7 Molecular determinants of failed resolution

Beneath the cellular circuits that may lock the synovium in a non-resolving state lies a smaller set of molecular switches that decide whether the resolution program can start at all. Resolution is an actively synthesized event with defined chemical and metabolic requirements (15), and the line between suppressing inflammation and enabling its resolution is drawn at exactly this molecular level (17). Three classes of determinant, namely lipid-mediator class switching, immunometabolic reprogramming, and epigenetic imprinting, each set a threshold that, in the rheumatoid synovium, appears biased toward non-resolution.

The most proximate determinant is failure of the lipid-mediator class switch that normally hands inflammation over to resolution. The molecular logic emerged when prostaglandin E2 was shown to reprogram eicosanoid biosynthesis and generate the lipoxin “stop” signal (16). In active RA, the handover looks incomplete. Pro-resolving mediators such as LXA4, RvD1 and RvE1 are proposed to be deficient in active disease. That would point to a synthetic or catabolic failure rather than only an excess of pro-inflammatory drive. Direct measurements of these mediators in human RA tissue remain limited, however (17).

The energetics of the tissue compound the lipid deficit, since the class switch and its downstream effector functions are metabolically demanding. The same aerobic glycolysis shift operates in rheumatoid fibroblast-like synoviocytes, and blocking it reduces their inflammation and invasion (49). This fits the broader principle that discrete metabolic modules program macrophage and stromal polarization (73). Succinate accumulation then stabilizes HIF-1α to sustain IL-1β production (74), a circuit reinforced by the hypoxic, HIF-1α–high milieu of the rheumatoid sublining (75). This pro-inflammatory metabolic setting counteracts the mitochondrial, itaconate-associated state that would allow resolution. The tissue’s bioenergetics may therefore withhold the substrate and reduce the power a productive class switch would need.

The most stable determinant is epigenetic, converting a transient inflammatory episode into a stably inherited non-resolving state, a molecular memory. The rheumatoid DNA-methylation signature (46) and the HOX-dependent positional memory that sets how fibroblasts at different sites respond to identical signals (48) both persist regardless of the initiating stimulus. They help explain why lowering cytokine load does not by itself reset the threshold of resolution. The molecular determinants stay fixed in a non-permissive configuration even when the cellular circuit is temporarily quieted. The architecture has three layers: a lipid switch that does not fire, a metabolic state that starves it, and an epigenetic imprint that locks the setting. Three mechanistically distinct entry points follow: restoring pro-resolving mediator tone, correcting the immunometabolic bias, and reversing the imprinted state.

Two issues remain unanswered. The first is whether these determinants are hierarchically ordered, for instance whether the metabolic state lies upstream of the epigenetic mark and is reversible before it. The second is whether any one class can be corrected alone, or whether the reinforcing links among them require co-targeting.

## 8 Difficult-to-treat RA through the resolution lens

### 8.1 Defining and stratifying refractory disease

The failed-resolution framework helps recast difficult-to-treat RA. D2T-RA does not qualify, on this basis, as a disorder of insufficient immunosuppression or treatment resistance in the usual sense. It refers to a synovium where the resolution program has failed to restart despite suppression of inflammatory output. The therapeutic question shifts from “how much suppression” to “which process”. D2T-RA becomes a natural experiment that separates dampening inflammation from enabling its resolution.

The EULAR definition of D2T-RA describes a treatment-defined stratum that has exhausted the current toolbox, though it is purposefully agnostic about mechanism. Failure of at least two biologic or targeted synthetic DMARDs with different mechanisms after inadequate response to conventional synthetic therapy is required. It also requires ongoing moderate-to-high disease activity and clinical features suggestive of active inflammation (13). Buch et al. defined persistent inflammatory refractory RA (PIRRA) in which inflammatory pathways remain active. In contrast, non-inflammatory refractory RA (NIRRA) is where symptoms persist despite resolution of overt synovitis (14). The failed-resolution lens explains PIRRA most clearly, a clinical problem in which inflammation will not be extinguished. This outcome is expected when the stromal–myeloid circuit is locked and the molecular determinants are set against resolution. NIRRA and refractoriness driven by comorbid conditions lie largely outside its scope.

Despite its heterogeneity, the D2T-RA population has a common treatment path. Drugs that target distinct nodes of the inflammatory cascade are cycled sequentially in patients: TNF (76, 77), IL-6 (78), JAK/STAT (79–81), T-cell co-stimulation (82), IL-1 (83), and B cells (84). None of these induces sustained remission. Pooled data from 23 studies with 27,987 patients found that 11.7% met EULAR criteria for difficult-to-treat disease (85). About half of those show evidence of persistent inflammatory refractory disease. The framework advanced here therefore addresses roughly 5% to 6% of all rheumatoid arthritis, a group carrying a disproportionate share of disability and healthcare burden.

Management guidance stresses optimizing adherence, controlling comorbidity, and trying different mechanistic classes (86). Once those are addressed, the available drugs run shallow. A systematic review of DMARD inefficacy in D2T-RA set out the immune and non-immune mechanisms that may underlie persistent disease (87). These features align with the stromal–myeloid circuit rather than with the targets of current biologics. Cycling through multiple immune checkpoints does not by itself flip the underlying switch, even though each agent suppresses a piece of the inflammatory network. The switch is proposed to be a circuit property, and its stability does not hinge on any single cytokine staying high.

Pathotype may also carry treatment implications. In a prospective biopsy cohort, twelve-week response to TNF blockade was substantially lower in patients with a pauci-immune synovium than in those with lymphoid or myeloid pathotypes. Only seven pauci-immune patients were assessed, however, and only one agent was tested (88).

### 8.2 Mechanistic mismatch: suppression is not resolution

Why does sequential escalation fail in a subset of patients? We propose a mismatch between how current drugs act and the biological state they meet. Approved DMARDs are not narrow cytokine sponges. Several have effects that extend into resolution biology itself, and a truthful version of the argument must begin there.

Methotrexate is the clearest case. In one cohort, response tracked with the frequency of CD39-expressing regulatory T cells. Cells from non-responder patients carried less CD39, generated less adenosine, and suppressed less effectively. In mice, blocking CD39 reversed the antiarthritic action of methotrexate, placing the regulatory arm on the causal pathway (89). TNF blockade comes closest to our own theme. Out of five biologics assessed on macrophages derived from people with active disease, only the agents targeting TNF repolarized the macrophages. CD40 and CD80 fell, while CD16, CD163, and MerTK rose, along with phagocytic capacity. The switch required early IL-10 and depended on STAT3, with negative feedback through SOCS3 and Gas6 (90). MerTK and Gas6 are the same receptor and ligand identified above as defective in rheumatoid efferocytosis.

Adalimumab promotes binding of membrane TNF to TNF-RII and expands functional FoxP3^+^ regulatory T (Treg) cells, unlike etanercept (91). JAK inhibitors act directly on synovial fibroblasts, not on lymphocytes alone, lowering their IL-6 output and their responsiveness to IL-6 trans-signaling (92). The strongest evidence comes from human tissue. Early RA synovium was imaged before treatment and 6 months later. A network of resident macrophages, identified as LYVE1^+^CD206^+^, was disrupted during the active disease and re-established in responders to traditional therapy. Chemokine, annexin, and TAM receptor pathways, including MERTK, were involved in recovery (93).

These findings sharpen the argument rather than overturn it. Three qualifications matter. None of these agents were intended to restore resolution, and the pro-resolving effects were discovered afterwards. No clinical trial has used a resolution readout as an endpoint, so we lack data on the size and durability of these effects in patients. Most of the evidence comes from *in vitro* studies, animal models, or comparisons between responders and non-responders after the fact. The precise claim is therefore narrower, and harder to dismiss, than a flat contradiction between suppression and resolution. Resolution is an axis that current drugs already touch, without aiming at it and without measuring it. In difficult-to-treat diseases, that incidental reach is clearly insufficient, and there is an argument for resolving an explicit target and an explicit readout.

What these drugs do not do becomes visible in refractory disease. None of them directly restore depleted pro-resolving macrophage populations, repair the failed lipid-mediator class switch, or reverse the epigenetic imprint that holds fibroblasts in an activated state. In anti-TNF inadequate responders, baseline synovial pathotype predicted differential response to rituximab versus tocilizumab (94). Multi-omics profiling of patients refractory to several targeted therapies found non-responders enriched for a stromal and fibroblast signature, and depleted for the inflammatory myeloid features those drugs address (12). Bone-directed treatment makes the same point. Denosumab inhibits structural progression without reducing inflammation or improving disease activity scores (95). A downstream effector of the loop can therefore be severed while the loop itself stays intact.

Read through the resolution lens, the therapeutic ceiling in refractory disease is not a failure of potency. It is a failure of relevance, and of measurement. The targets sit on the inflammatory arm, while the disease persists because the resolution arm stays absent. Treat-to-target strategies push escalation toward maximal suppression (3, 96, 97). If the deficit is lost resolution competence instead of excess inflammatory drive, more suppression cannot close the gap. A subset of D2T patients may need a different strategy. The aim would be to restore missing functions: re-arming pro-resolving macrophages, correcting the lipid-mediator deficit, reprogramming or eliminating pathological fibroblasts, and breaking the feed-forward circuit at its structural anchors. Which fraction of refractory disease is mechanistically resolution-incompetent, whether that subset can be identified prospectively, and whether the window for restoration closes with disease duration all remain open.

## 9 Resolution-restoring strategies: mechanism and clinical evidence

**Figure 3:**
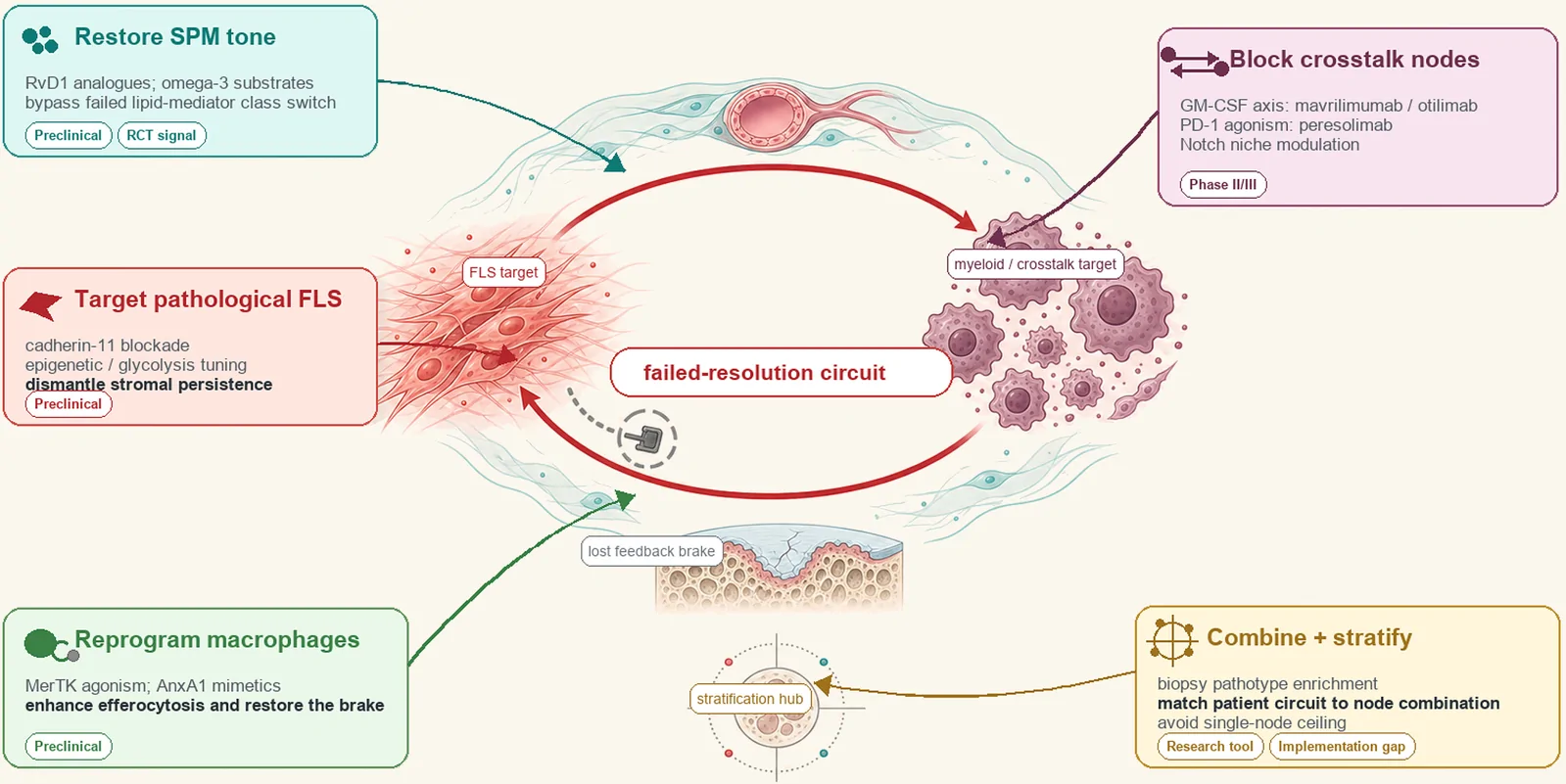
A resolution-restoring therapeutic map: five strategy arms aligned to the nodes of the failed-resolution circuit. A simplified rendering of the circuit in **Figure 2** (fibroblast↔myeloid loop, pro-resolving brake, and spatial niche) sits at the center as a hub, with five strategy arms radiating to the node each addresses; representative agents are shown in italics and maturity is indicated per arm (clinical readout/early-phase/preclinical), keyed to **Table 3**. (i) Restoring SPM tone (resolvin D1 and analogs; ω-3 substrate) addresses the lipid-mediator effector deficit. (ii) Macrophage reprogramming (MerTK agonism, annexin A1 mimetics, efferocytosis enhancers) targets the lost negative-feedback brake. (iii) Fibroblast targeting (cadherin-11 blockade, epigenetic reversal, glycolysis interference) acts on the stromal pole. (iv) Crosstalk-node blockade acts on the connecting arms and spatial niche: GM-CSF-axis blockade (mavrilimumab, otilimab), PD-1 agonism to re-arm the inhibitory brake (peresolimab), and Notch-niche disruption. (v) Combination and stratification encircles the whole model, reflecting that a redundant circuit requires multi-node interruption with biopsy-/pathotype-guided patient selection. Each strategy anchors to a distinct circuit node rather than forming a parallel list, and single-node interventions such as GM-CSF blockade face a ceiling set by circuit redundancy. AnxA1, annexin A1; GM-CSF, granulocyte–macrophage colony-stimulating factor; MerTK, MER tyrosine kinase; PD-1, programmed cell death protein 1; SPM, specialized pro-resolving mediator. Figure created with BioRender.com.

Restoring resolution in difficult-to-treat rheumatoid arthritis calls for a different therapeutic logic from intensifying immunosuppression. The aim is not to add another brake to inflammatory signaling. It is to re-enable the endogenous processes that failed: to restock the depleted mediators, reprogram the cells, and dismantle the circuits that oppose resolution. Five complementary axes follow, each addressing a distinct mechanistic deficit identified above: (i) restoring specialized pro-resolving mediator (SPM) tone; (ii) reprogramming macrophages toward pro-resolving phenotypes and enhancing efferocytosis; (iii) targeting or reprogramming pathological fibroblast subsets; (iv) interrupting the stromal–myeloid crosstalk nodes that sustain the feed-forward circuit; and (v) combining these approaches to reflect the redundancy and mutual reinforcement of the underlying biology (17, 23). The evidence behind them is unevenly distributed. Each axis is set out below with its mechanism and, where one exists, its clinical readout (**Figure 3**, **Table 3**).

**Table 3:** Resolution-restoring therapeutic candidates in rheumatoid arthritis: target, mechanism, development stage, and representative evidence.

Strategy arm	Agent/approach	Molecular target	Proposed resolution mechanism	Highest development stage	Representative evidence & outcome	Ref.
SPM tone	Resolvin D1/analog	ALX/FPR2	Replenish lipid mediators; restart downstream class-switch	Preclinical	Anti-inflammatory and chondroprotective in preclinical inflammatory arthritis	(98)
SPM tone	Fish oil/ω-3 substrate	SPM precursor substrate	Increase substrate availability; raise resolution threshold	RCT (recent-onset RA)	Adjunct to algorithmic DMARD therapy; modest increase in remission	(99)
Macrophage reprogramming	MerTK agonism/protein S	MerTK	Enhance efferocytosis; restore resolution-phase macrophage identity	Preclinical	Mechanistic validation; not yet in registrational RA trials	(60)
Macrophage reprogramming	AnxA1 mimetic	FPR2 (JAK/STAT/SOCS)	Suppress monocyte activation; glucocorticoid-independent resolution	Preclinical	Arthritis models; mediator of GC-dependent resolution	(61, 100)
Fibroblast targeting	Cadherin-11 blockade	Cadherin-11	Disrupt lining formation/invasive phenotype	Preclinical (not validated in RA)	Reduced inflammation and lining disruption in models	(43)
Fibroblast targeting	Epigenetic/glycolysis intervention	DNMT/glycolytic flux	Reverse imprint; remove metabolic maintenance	Preclinical (speculative)	Glycolysis blockade lowers FLS inflammation/invasion	(49)
Crosstalk-node blockade	Mavrilimumab	GM-CSFRα	Interrupt the stroma→myeloid survival arm	Phase IIb (EARTH EXPLORER 1)	Met primary endpoint; reduced disease activity	(101)
Crosstalk-node blockade	Otilimab	GM-CSF	As above (second antibody)	Phase III (contRAst 1/2/3)	Superior to placebo but not to active comparator; discontinued for efficacy ceiling; acceptable safety	(24, 102, 103)
Crosstalk-node blockade	Peresolimab	PD-1 (agonism)	Re-arm the endogenous inhibitory arm (re-arm the brake)	Phase II	Met primary endpoint; proof-of-concept	(104)
Crosstalk-node blockade	Notch-niche intervention	NOTCH3 niche	Disrupt spatial anchoring; destabilize the circuit	Preclinical (speculative)	Niche-construction mechanistic evidence	(42)
Combination-stratification	Biopsy pathotype/molecular stratification enrichment	—	Identify the resolution-incompetent subgroup; mechanism-matched allocation	Research tool (not a clinical-grade diagnostic)	R4RA/AMP II demonstrate feasibility; biomarker to be validated	(12, 20, 94)

### 9.1 Restoring pro-resolving mediator tone

The first axis addresses the lipid-mediator deficit head-on, supplementing or mimicking SPMs to bypass the failed endogenous class switch. Resolvin D1 reduces joint inflammation and protects cartilage through ALX/FPR2 receptor signaling in preclinical models of inflammatory arthritis (98). A stable SPM analog can therefore recapitulate the resolution endpoint even when the tissue’s own synthetic machinery is impaired. More broadly accessible is dietary provision of SPM precursors. Fish oil is rich in the omega-3 polyunsaturated fatty acids that serve as SPM substrate. In a randomized trial of recent-onset RA, omega-3 raised remission rates when added to an algorithm-based DMARD regimen (99). The signal is modest, but it indicates that substrate availability can change the resolution threshold. No stable SPM analog has yet reached phase II or III in RA. Resolvin D1 and its analogs are under study in other inflammatory conditions, which makes them high-priority candidates once formulation and stability are resolved.

### 9.2 Reprogramming the macrophage compartment

The second axis focuses on macrophages to restore the MerTK^+^/TREM2^hi^ pro-resolving populations depleted in persistent disease. MerTK couples efferocytosis to anti-inflammatory reprogramming, which makes it a direct leverage point. Strengthening MerTK signaling enhances continual efferocytosis and a resolution-phase macrophage identity (60). Candidate routes include agonist antibodies, provision of its bridging ligand protein S, and metabolic modification. The annexin A1 (AnxA1) pathway plays a complementary role, acting through formyl peptide receptors on monocytes (61). Its role in mediating glucocorticoid-driven resolution in arthritis models (100) casts it as a glucocorticoid-independent resolution agonist. Such an agonist could lower the steroid burden in D2T-RA by recreating the resolving arm without the immunosuppressive cost. MerTK agonism, AnxA1 mimetics, and efferocytosis enhancers are validated in preclinical resolution biology, but none is in a registered RA trial.

### 9.3 Targeting pathological fibroblasts

The third axis targets the pathological fibroblast: selective depletion, phenotype reprogramming, or blockade of the niche signals that maintain its pathogenic state. Cadherin-11 is a validated structural target. Genetic or pharmacological blockade disrupts lining formation and diminishes inflammation in models (43). Remedying the glycolytic skew (49) or reversing the DNA methylation imprint are more speculative but mechanistically plausible routes to fibroblast reprogramming. Clinically, this axis is the least advanced. Cadherin-11 blockades, metabolic correction, and epigenetic reprogramming have not been tested in RA trials. Two hurdles stand out. Depleting or reprogramming fibroblasts selectively, without impairing normal stromal functions, is pharmacologically non-trivial. The absence of a validated fibroblast-derived biomarker also makes patient selection speculative.

### 9.4 Interrupting stromal–myeloid crosstalk nodes

The fourth axis interrupts the crosstalk nodes that couple stroma to myeloid cells, and it is the most clinically advanced. Blocking GM-CSF tests the fibroblast-to-myeloid survival arm directly. Mavrilimumab, a monoclonal antibody against GM-CSF receptor-α, met its primary endpoint in the phase IIb EARTH EXPLORER 1 trial, with significant reductions in disease activity in methotrexate-inadequate responders (101). That signal prompted the otilimab program, a second anti-GM-CSF antibody tested in three phase III trials (contRAst 1, 2, and 3). These enrolled patients with inadequate response to conventional, biologic, or targeted synthetic DMARDs (102). In contRAst 1 and 2, otilimab was superior to placebo on ACR20 but inferior to the active comparator tofacitinib. In the more refractory contRAst 3 population, it failed to separate from placebo on the ACR20 primary endpoint, and it did not demonstrate non-inferiority to sarilumab (24). A long-term extension (103) confirmed acceptable safety, yet the program was terminated for an efficacy ceiling rather than toxicity. GM-CSF blockades can therefore suppress disease activity, so the arm is real and therapeutically accessible. In heavily pre-treated D2T patients, however, removing one node of a redundant crosstalk network appears insufficient to restore resolution durably, which is what the circuit model would predict.

A conceptually distinct crosstalk modulator is the programmed cell death protein 1 (PD-1) agonist peresolimab. Instead of blocking an activating signal, it amplifies an endogenous checkpoint through the PD-1 inhibitory receptor on T cells and myeloid cells. This reestablishes an inhibitory tone that was lost or overridden. That is an analogy to resolution rather than an established resolution mechanism. In a phase II trial in active RA, peresolimab demonstrated statistically significant and clinically meaningful reductions in disease activity and met its primary endpoint (104). This is consistent with the failed-resolution framework prediction of “re-arm the brake” logic. But this is not proof. The readout is early, and durability, safety in broader populations, and positioning against standard-of-care are still undefined. The evidence is proof of concept: restoring inhibitory tone rather than intensifying blockade of activators can alter the disease state. The Notch niche spatially organizes the perivascular fibroblast and myeloid compartments (42). It is a speculative but mechanistically compelling target, since disrupting niche architecture could destabilize the circuit at its spatial anchor.

### 9.5 Combination strategies, stratification, and trial design

The fifth axis is synthesis. If the failed-resolution state is held in place by redundant, mutually reinforcing mechanisms, single-axis interventions are unlikely to flip the switch durably in most D2T patients. Combinations could be synergistic rather than merely additive when they hit distinct layers. Lipid mediators and macrophage reprogramming (axes i–ii) address the resolution effector machinery. Fibroblast targeting and crosstalk blockade (axes iii–iv) dismantle the circuit that opposes it. The toolkit therefore spans early proof-of-principle (MerTK agonists and Notch inhibitors) and agents already at clinical readout (SPM precursors, GM-CSF blockade, and PD-1 agonism).

The central translational challenge is stratification: identifying which patients are locked in a resolution-incompetent state rather than in another difficult-to-treat mechanism, such as non-inflammatory refractory disease or comorbidity-driven pseudo-refractoriness. Synovial biopsy pathotyping (94) and transcriptomic profiling (12) show that such stratification is biologically feasible since fibroid- or stromal-high patients exist and can be identified. These remain research tools, not clinical-grade diagnostics. Until validated circulating or imaging biomarkers can approximate the molecular state non-invasively, trial populations will stay heterogeneous and effect sizes diluted. Trial design for combinations is a second bottleneck. Testing SPM supplementation plus macrophage reprogramming plus crosstalk blockade in a factorial or adaptive platform demands infrastructure, biomarker-driven enrichment, and regulatory appetite that are not yet in place. The GM-CSF monotherapy ceiling nonetheless argues that such designs are necessary. The clearest opportunity is early intervention: testing resolution-restoring agents in recent-onset or early refractory disease before ectopic lymphoid structures and durable epigenetic imprints lock the circuit architecturally.

## 10 Limitations, conflicting evidence, and future directions

The framework set out here is a hypothesis, not a definitive explanation. Before it can guide treatment, three things need to be stated openly: what the present evidence cannot show, where findings are at odds, and what still separates the concept from the clinic.

### 10.1 Limitations of the current evidence base

The failed-resolution switch is a model. No study has yet disabled one arm of the circuit in human synovium and shown that resolution returns. Its components rest on separate lines of work, assembled here into a single scheme, and that assembly is an interpretation rather than a result.

Most of the evidence is cross-sectional. Atlases capture only the moment the tissue was sampled, showing which cells sit together in inflamed synovium but not which arrived first. Cluster boundaries also depend on analytical choices: no agreed rule fixes the number of clusters, and automated annotation inherits the biases of the reference dataset used. Sequencing-based spatial platforms currently resolve several cells per spot rather than single cells, so identity is assigned by deconvolution (105).

Sample handling introduces further distortion. Warm enzymatic dissociation induces heat-shock and immediate-early genes, creating a stress-activated population that was not present in the tissue (106, 107). Cryopreservation depletes some populations, and single-nucleus libraries under-represent lymphocytes (108). Subset frequencies measured this way are partly a property of the protocol.

Receptor-ligand analyses inherit the same constraints. The signaling axes are inferred from co-expression using computational frameworks, and most have not been tested experimentally (109). The macrophage-derived HBEGF axis is the exception, validated by coculture and by pharmacological blockade (66). Whether the cells described here cause synovial inflammation or accompany it remains open (110). Settling that will need different experiments: lineage tracing, conditional deletion in the relevant compartments, paired longitudinal biopsies, and human coculture or organoid systems that rebuild the circuit (105, 111).

### 10.2 Conflicting evidence and heterogeneity

Four areas are less settled than the sections above may imply.

Macrophage subset definitions do not align across studies. MerTK^+^TREM2^hi^ populations (22), CX3CR1^+^ barrier macrophages (21), and independently derived schemes such as IL-1B^+^CCL20^+^ and SPP1^+^MT2A^+^ clusters (112) were each defined in different cohorts with different markers. A reanalysis integrating five synovial datasets recovered no cluster marked specifically by TREM2, finding TREM2 expression spread across several myeloid subsets instead. The authors attributed these differences to variations in patient recruitment and clustering workflow (113). *In situ* staining in the same study still identified TREM2^+^ macrophages as a distinct population. The dispute is over the definition of subsets, not over the existence of cells. Frequencies reported in one cohort should therefore not be read as directly comparable with another.

Findings of clinical studies on pro-resolving substrates are inconsistent. A meta-analysis of 23 placebo-controlled trials produced confidence intervals crossing the null for pain, tender joints, swollen joints, and C-reactive protein. It rated the certainty of that evidence as low or very low. The same analysis found that NSAID sparing seen in earlier reports may have been biased by inadequate blinding (114). A further meta-analysis found clear biochemical target engagement without any significant changes in ESR, CRP or DAS28 (115). The positive adjunctive trial noted above must be read against this background (99).

Whether these mediators can be reliably measured in human blood is itself disputed. One group holds that reported peaks fall below any credible limit of detection once the full baseline is considered. The same critique reports that confirmatory fragmentation spectra do not match authentic standards, and that solvent blanks produce the same diagnostic ions (116). The authors of the rheumatoid biomarker study at issue (117) reject that reading, attributing the critics’ signals to instrument contamination (118). Independent method development has reported these mediators to be generally not quantifiable in plasma or serum (119). A human study also found them largely undetectable after fish oil, despite clear substrate delivery (120). The proposal that pro-resolving tone is deficient in rheumatoid arthritis should be read with this dispute in view.

Rodent arthritis does not reproduce chronic non-resolution. Antigen-induced arthritis resolves within about 5 days, and serum-transfer arthritis within about twenty. Collagen-induced arthritis begins resolving from day 10 to 14, ending in fibrosis and repair not seen in human disease (121). Models with genuine chronic disease are scarce, and passive-transfer models lack endogenous T and B cell autoimmunity altogether (122). The phenotype at the center of this review, a synovium that will not resolve, is precisely what these models lack. Conclusions drawn from them about resolution-restoring interventions therefore carry unusual uncertainty.

Difficult-to-treat disease is not a single entity. Fibromyalgia, depression, and obesity are each independently associated with the label (123, 124), and in one cohort, 43% of difficult-to-treat patients showed no evidence of active inflammation upon ultrasound (125). Drug intolerance supplies a further route in (126). The framework developed here applies to the inflammatory fraction, roughly 5% to 6% of all rheumatoid arthritis. Applied to the label as a whole, it would be a category error.

### 10.3 Barriers to translation

Pro-resolving mediators meet chemical obstacles before clinical ones. Resolvin D1 is inactivated enzymatically by eicosanoid oxidoreductase, and oxidation of its C17 alcohol removes activity, while the aspirin-triggered 17R epimer is metabolized more slowly (127). Analogs built to resist 15-prostaglandin dehydrogenase retain pro-resolving activity *in vivo*, which shows the problem is tractable rather than solved (128). Polyunsaturated lipids are oxidation-prone, so stereochemical purity, batch consistency, and storage become manufacturing constraints.

Patient selection is the harder barrier. Biopsy-driven trials are feasible, and two randomized programs have now run at scale (94, 129). Turning tissue phenotype into a treatment decision has not yet worked. In R4RA, machine-learning predictors reached areas under the curve of 0.68 to 0.74, which the authors judged too low for direct clinical use without further validation (12). STRAP then tested the approach prospectively. Among B cell-poor patients, 16-week ACR20 was 60% with etanercept or tocilizumab and 59% with rituximab, and the binary classification did not predict response to B cell depletion (129). Synovial pathotyping thus remains a research tool rather than a qualified biomarker, and formal qualification is a demanding regulatory path (130). Across rheumatology more widely, treatment choice still rests largely on trial and error (131).

Endpoints pose a third problem. Regulatory guidance and trial practice in rheumatoid arthritis rest on composite disease-activity measures, patient-reported outcomes and structural damage (132). These do not track resolution. One imaging study examined patients in DMARD-induced remission who were asymptomatic with clinically normal joints. On MRI, 96% had synovitis and 46% had bone marrow edema. Ultrasound showed grey-scale synovial hypertrophy in 73% and increased power Doppler signal in 43% (133). Remission defined by DAS28 also tolerates residual activity by construction (134). Outcomes can dissociate in the other direction too. Inhibiting bone destruction need not track with control of joint inflammation, as anti-RANKL therapy showed (95). No endpoint in current use reports directly on whether the capacity to resolve has been restored.

The history of the field urges caution about combination strategies. A circuit with redundant arms invites blockade at more than one node, yet earlier attempts to combine immunomodulatory agents in rheumatoid arthritis were not rewarded. Adding anakinra to etanercept gave no added efficacy over etanercept alone and increased serious infections (135). Abatacept combined with background biologic treatment increased serious adverse events from approximately 12% to 22%, and the investigators advised against the combination (136). Any multi-node effort to restore resolution will have to surpass that threshold.

### 10.4 A sequential research agenda

These gaps establish an agenda with sequential steps: each question relies on the previous answer.

The first question is whether resolution can be measured. Lipid-mediator signatures are the most direct, although their analytical basis must first be established for the reasons above. Pro-resolving synovial macrophage states track with sustained remission and provide a tissue correlation that could be followed over time (22). Molecular pathotyping is the most mature approach, classifying patients into subgroups with diverse treatment responses based on cell-type abundance (12, 20). Before any of them can be said to report resolution rather than inflammatory burden, each needs prospective validation against longitudinal outcomes.

The second is whether patients can be selected on that basis. A coarse binary classification is not enough (129). Finer stratification, mapped onto the inflammatory and non-inflammatory axes of refractory disease (14), would need to be built into trial design and tested prospectively rather than recovered *post hoc*.

The third is whether restoration can be shown. This requires endpoints that index resolution, models of chronic non-resolution, and longitudinal profiling of treated tissue. Recent longitudinal profiling illustrates the point (54). An activity score alone reveals little about a shift of that kind. Measuring, selecting, and proving resolution would together replace the suppression-centered paradigm that has shaped therapeutic development so far.

## 11 Conclusion

The persistence of difficult-to-treat rheumatoid arthritis is not, we have argued, simply inflammation that is too strong or too drug-resistant. It is better understood as the active failure of the program meant to bring inflammation to a close. We suggest that the bidirectional crosstalk of synovial fibroblasts and myeloid cells holds it in place, with lipid-mediator, metabolic, and epigenetic switches sustaining it. When viewed this way, difficult-to-treat disease is less a descriptive clinical label than an actionable mechanistic coordinate. There are four consequences. It could explain why increased immunosuppression fails. It could produce testable hypotheses regarding defined crosstalk circuits. It could supply resolution-capacity readouts for patient stratification, among them lipid signatures, pro-resolving macrophage states, and synovial pathotypes. And it could move therapeutic development from the intensity of inflammation toward the nodes that restore its resolution.

This is not a claim that current drugs act only by suppression. Several already reach pro-resolving pathways. The point is that these effects were neither designed for nor measured, and in refractory disease they are plainly insufficient. The account speaks most directly to the persistent-inflammatory form of refractory disease. Symptom persistence driven by non-inflammatory mechanisms or comorbidity lies outside its scope and needs different tools.

Whether that transition happens depends on the agenda above. Each advance must be allowed to test the framework, not merely to illustrate it. Validated readouts and mechanism-enriched trials could confirm the failed-resolution account. They could equally show that suppression and resolution are less separable in human synovium than this model supposes. Either outcome would be progress.

## References

[1] Smolen JS Aletaha D Barton A Burmester GR Emery P Firestein GS . Rheumatoid arthritis. Nat Rev Dis Primers. (2018) 4:18001. doi: 10.1038/nrdp.2018.1 29417936

[2] McInnes IB Schett G . The pathogenesis of rheumatoid arthritis. N Engl J Med. (2011) 365:2205–19. doi: 10.1056/NEJMra1004965 22150039

[3] Grigor C Capell H Stirling A McMahon AD Lock P Vallance R . Effect of a treatment strategy of tight control for rheumatoid arthritis (the TICORA study): a single-blind randomised controlled trial. Lancet. (2004) 364:263–9. doi: 10.1016/S0140-6736(04)16676-2 15262104

[4] McInnes IB Schett G . Cytokines in the pathogenesis of rheumatoid arthritis. Nat Rev Immunol. (2007) 7:429–42. doi: 10.1038/nri2094 17525752

[5] Schellekens GA de Jong BA van den Hoogen FH van de Putte LB van Venrooij WJ . Citrulline is an essential constituent of antigenic determinants recognized by rheumatoid arthritis-specific autoantibodies. J Clin Invest. (1998) 101:273–81. doi: 10.1172/JCI1316 9421490 PMC508564

[6] Schellekens GA Visser H de Jong BA van den Hoogen FH Hazes JM Breedveld FC . The diagnostic properties of rheumatoid arthritis antibodies recognizing a cyclic citrullinated peptide. Arthritis Rheum. (2000) 43:155–63. doi: 10.1002/1529-0131(200001)43:1<155::AID-ANR20>3.0.CO;2-3 10643712

[7] Aletaha D Neogi T Silman AJ Funovits J Felson DT Bingham C . 2010 rheumatoid arthritis classification criteria: an American College of Rheumatology/European League Against Rheumatism collaborative initiative. Ann Rheum Dis. (2010) 69:1580–8. doi: 10.1136/ard.2010.138461 20699241

[8] Nielen MM van Schaardenburg D Reesink HW van de Stadt RJ van der Horst-Bruinsma IE de Koning MH . Specific autoantibodies precede the symptoms of rheumatoid arthritis: a study of serial measurements in blood donors. Arthritis Rheum. (2004) 50:380–6. doi: 10.1002/art.20018 14872479

[9] Rantapaa-Dahlqvist S de Jong BA Berglin E Hallmans G Wadell G Stenlund H . Antibodies against cyclic citrullinated peptide and IgA rheumatoid factor predict the development of rheumatoid arthritis. Arthritis Rheum. (2003) 48:2741–9. doi: 10.1002/art.11223 14558078

[10] Elliott MJ Maini RN Feldmann M Kalden JR Antoni C Smolen JS . Randomised double-blind comparison of chimeric monoclonal antibody to tumour necrosis factor alpha (cA2) versus placebo in rheumatoid arthritis. Lancet. (1994) 344:1105–10. doi: 10.1016/s0140-6736(94)90628-9 7934491

[11] Maini R St Clair EW Breedveld F Furst D Kalden J Weisman M . Infliximab (chimeric anti-tumour necrosis factor alpha monoclonal antibody) versus placebo in rheumatoid arthritis patients receiving concomitant methotrexate: a randomised phase III trial. ATTRACT Study Group. Lancet. (1999) 354:1932–9. doi: 10.1016/s0140-6736(99)05246-0 10622295

[12] Rivellese F Surace AEA Goldmann K Sciacca E Cubuk C Giorli G . Rituximab versus tocilizumab in rheumatoid arthritis: synovial biopsy-based biomarker analysis of the phase 4 R4RA randomized trial. Nat Med. (2022) 28:1256–68. doi: 10.1038/s41591-022-01789-0 35589854 PMC9205785

[13] Nagy GR Roodenrijs NM Welsing PM Kedves M Hamar A van der Goes MC . EULAR definition of difficult-to-treat rheumatoid arthritis. Ann Rheum Dis. (2021) 80:31–5. doi: 10.1136/annrheumdis-2020-217344 33004335 PMC7788062

[14] Buch MH Eyre S McGonagle D . Persistent inflammatory and non-inflammatory mechanisms in refractory rheumatoid arthritis. Nat Rev Rheumatol. (2020) 17:17–33. doi: 10.1038/s41584-020-00541-7 33293696

[15] Serhan CN . Pro-resolving lipid mediators are leads for resolution physiology. Nature. (2014) 510:92–101. doi: 10.1038/nature13479 24899309 PMC4263681

[16] Levy BD Clish CB Schmidt B Gronert K Serhan CN . Lipid mediator class switching during acute inflammation: signals in resolution. Nat Immunol. (2001) 2:612–9. doi: 10.1038/89759 11429545

[17] Fullerton JN Gilroy DW . Resolution of inflammation: a new therapeutic frontier. Nat Rev Drug Discov. (2016) 15:551–67. doi: 10.1038/nrd.2016.39 27020098

[18] Croft AP Campos J Jansen K Turner JD Marshall J Attar M . Distinct fibroblast subsets drive inflammation and damage in arthritis. Nature. (2019) 570:246–51. doi: 10.1038/s41586-019-1263-7 31142839 PMC6690841

[19] Zhang F Wei K Slowikowski K Fonseka CY Rao DA Kelly S . Defining inflammatory cell states in rheumatoid arthritis joint synovial tissues by integrating single-cell transcriptomics and mass cytometry. Nat Immunol. (2019) 20:928–42. doi: 10.1038/s41590-019-0378-1 31061532 PMC6602051

[20] Zhang F Jonsson AH Nathan A Millard N Curtis M Xiao Q . Deconstruction of rheumatoid arthritis synovium defines inflammatory subtypes. Nature. (2023) 623:616–24. doi: 10.1038/s41586-023-06708-y 37938773 PMC10651487

[21] Culemann S Gruneboom A Nicolas-Avila JA Weidner D Lammle KF Rothe T . Locally renewing resident synovial macrophages provide a protective barrier for the joint. Nature. (2019) 572:670–5. doi: 10.1038/s41586-019-1471-1 31391580 PMC6805223

[22] Alivernini S MacDonald L Elmesmari A Finlay S Tolusso B Gigante MR . Distinct synovial tissue macrophage subsets regulate inflammation and remission in rheumatoid arthritis. Nat Med. (2020) 26:1295–306. doi: 10.1038/s41591-020-0939-8 32601335

[23] Nygaard G Firestein GS . Restoring synovial homeostasis in rheumatoid arthritis by targeting fibroblast-like synoviocytes. Nat Rev Rheumatol. (2020) 16:316–33. doi: 10.1038/s41584-020-0413-5 32393826 PMC7987137

[24] Taylor PC Weinblatt ME McInnes IB Atsumi T Strand V Takeuchi T . Anti-GM-CSF otilimab versus sarilumab or placebo in patients with rheumatoid arthritis and inadequate response to targeted therapies: a phase III randomised trial (contRAst 3). Ann Rheum Dis. (2023) 82:1527–37. doi: 10.1136/ard-2023-224449 37696589 PMC10646837

[25] Serhan CN Chiang N Dalli J . The resolution code of acute inflammation: novel pro-resolving lipid mediators in resolution. Semin Immunol. (2015) 27:200–15. doi: 10.1016/j.smim.2015.03.004 25857211 PMC4515371

[26] Serhan CN Yang R Martinod K Kasuga K Pillai PS Porter TF . Maresins: novel macrophage mediators with potent antiinflammatory and proresolving actions. J Exp Med. (2009) 206:15–23. doi: 10.1084/jem.20081880 19103881 PMC2626672

[27] Basil MC Levy BD . Specialized pro-resolving mediators: endogenous regulators of infection and inflammation. Nat Rev Immunol. (2015) 16:51–67. doi: 10.1038/nri.2015.4 26688348 PMC5242505

[28] Serhan CN Chiang N Dalli J Levy BD . Lipid mediators in the resolution of inflammation. Cold Spring Harb Perspect Biol. (2014) 7:a016311. doi: 10.1101/cshperspect.a016311 25359497 PMC4315926

[29] Clish CB O'Brien JA Gronert K Stahl GL Petasis NA Serhan CN . Local and systemic delivery of a stable aspirin-triggered lipoxin prevents neutrophil recruitment *in vivo*. Proc Natl Acad Sci USA. (1999) 96:8247–52. doi: 10.1073/pnas.96.14.8247 10393980 PMC22220

[30] Ohira T Arita M Omori K Recchiuti A Van Dyke TE Serhan CN . Resolvin E1 receptor activation signals phosphorylation and phagocytosis. J Biol Chem. (2010) 285:3451–61. doi: 10.1074/jbc.M109.044131 19906641 PMC2823415

[31] Yurdagul A Subramanian M Wang X Crown SB Ilkayeva OR Darville L . Macrophage metabolism of apoptotic cell-derived arginine promotes continual efferocytosis and resolution of injury. Cell Metab. (2020) 31:518–533.e10. doi: 10.1016/j.cmet.2020.01.001 32004476 PMC7173557

[32] Serhan CN Dalli J Karamnov S Choi A Park CK Xu ZZ . Macrophage proresolving mediator maresin 1 stimulates tissue regeneration and controls pain. FASEB J. (2012) 26:1755–65. doi: 10.1096/fj.11-201442 22253477 PMC3316905

[33] Fredman G Serhan CN . Specialized pro-resolving mediators in vascular inflammation and atherosclerotic cardiovascular disease. Nat Rev Cardiol. (2024) 21:808–23. doi: 10.1038/s41569-023-00984-x 38216693 PMC12863063

[34] Gilroy DW Lawrence T Perretti M Rossi AG . Inflammatory resolution: new opportunities for drug discovery. Nat Rev Drug Discov. (2004) 3:401–16. doi: 10.1038/nrd1383 15136788

[35] Perretti M Leroy X Bland EJ Montero-Melendez T . Resolution pharmacology: opportunities for therapeutic innovation in inflammation. Trends Pharmacol Sci. (2015) 36:737–55. doi: 10.1016/j.tips.2015.07.007 26478210

[36] Firestein GS . Evolving concepts of rheumatoid arthritis. Nature. (2003) 423:356–61. doi: 10.1038/nature01661 12748655

[37] Humby F Bombardieri M Manzo A Kelly S Blades MC Kirkham B . Ectopic lymphoid structures support ongoing production of class-switched autoantibodies in rheumatoid synovium. PloS Med. (2009) 6:e1. doi: 10.1371/journal.pmed.0060001 19143467 PMC2621263

[38] Khandpur R Carmona-Rivera C Vivekanandan-Giri A Gizinski A Yalavarthi S Knight JS . NETs are a source of citrullinated autoantigens and stimulate inflammatory responses in rheumatoid arthritis. Sci Transl Med. (2013) 5:178ra40. doi: 10.1126/scitranslmed.3005580 23536012 PMC3727661

[39] Schett G Gravallese E . Bone erosion in rheumatoid arthritis: mechanisms, diagnosis and treatment. Nat Rev Rheumatol. (2012) 8:656–64. doi: 10.1038/nrrheum.2012.153 23007741 PMC4096779

[40] Bottini N Firestein GS . Duality of fibroblast-like synoviocytes in RA: passive responders and imprinted aggressors. Nat Rev Rheumatol. (2013) 9:24–33. doi: 10.1038/nrrheum.2012.190 23147896 PMC3970924

[41] Mizoguchi F Slowikowski K Wei K Marshall JL Rao DA Chang SK . Functionally distinct disease-associated fibroblast subsets in rheumatoid arthritis. Nat Commun. (2018) 9:789. doi: 10.1038/s41467-018-02892-y 29476097 PMC5824882

[42] Wei K Korsunsky I Marshall JL Gao A Watts GFM Major T . Notch signalling drives synovial fibroblast identity and arthritis pathology. Nature. (2020) 582:259–64. doi: 10.1038/s41586-020-2222-z 32499639 PMC7841716

[43] Lee DM Kiener HP Agarwal SK Noss EH Watts GFM Chisaka O . Cadherin-11 in synovial lining formation and pathology in arthritis. Science. (2007) 315:1006–10. doi: 10.1126/science.1137306 17255475

[44] Kiener HP Lee DM Agarwal SK Brenner MB . Cadherin-11 induces rheumatoid arthritis fibroblast-like synoviocytes to form lining layers *in vitro*. Am J Pathol. (2006) 168:1486–99. doi: 10.2353/ajpath.2006.050999 16651616 PMC1606584

[45] Lefevre S Knedla A Tennie C Kampmann A Wunrau C Dinser R . Synovial fibroblasts spread rheumatoid arthritis to unaffected joints. Nat Med. (2009) 15:1414–20. doi: 10.1038/nm.2050 19898488 PMC3678354

[46] Whitaker JW Shoemaker R Boyle DL Hillman J Anderson D Wang W . An imprinted rheumatoid arthritis methylome signature reflects pathogenic phenotype. Genome Med. (2013) 5:40. doi: 10.1186/gm444 23631487 PMC3706831

[47] Nakano K Boyle DL Firestein GS . Regulation of DNA methylation in rheumatoid arthritis synoviocytes. J Immunol. (2013) 190:1297–303. doi: 10.4049/jimmunol.1202572 23277489 PMC3552038

[48] Frank-Bertoncelj M Trenkmann M Klein K Karouzakis E Rehrauer H Bratus A . Epigenetically-driven anatomical diversity of synovial fibroblasts guides joint-specific fibroblast functions. Nat Commun. (2017) 8:14852. doi: 10.1038/ncomms14852 28332497 PMC5376654

[49] Garcia-Carbonell R Divakaruni AS Lodi A Vicente-Suarez I Saha A Cheroutre H . Critical role of glucose metabolism in rheumatoid arthritis fibroblast-like synoviocytes. Arthritis Rheumatol. (2016) 68:1614–26. doi: 10.1002/art.39608 26815411 PMC4963240

[50] Pohlers D Beyer A Koczan D Wilhelm T Thiesen HJ Kinne RW . Constitutive upregulation of the transforming growth factor-beta pathway in rheumatoid arthritis synovial fibroblasts. Arthritis Res Ther. (2007) 9:R59. doi: 10.1186/ar2217 17594488 PMC2206335

[51] Frangogiannis NG . Transforming growth factor-β in tissue fibrosis. J Exp Med. (2020) 217:e20190103. doi: 10.1084/jem.20190103 32997468 PMC7062524

[52] Younesi FS Miller AE Barker TH Rossi FMV Hinz B . Fibroblast and myofibroblast activation in normal tissue repair and fibrosis. Nat Rev Mol Cell Biol. (2024) 25:617–38. doi: 10.1038/s41580-024-00716-0 38589640

[53] Watson RS Gouze E Levings PP Bush ML Kay JD Jorgensen MS . Gene delivery of TGF-β1 induces arthrofibrosis and chondrometaplasia of synovium *in vivo*. Lab Invest. (2010) 90:1615–27. doi: 10.1038/labinvest.2010.145 20697373 PMC3724510

[54] Bhamidipati K McIntyre ABR Kazerounian S Ce G Wong SW Tran M . Spatial patterning of fibroblast TGFβ signaling underlies treatment resistance in rheumatoid arthritis. Nat Immunol. (2026) 27:556–71. doi: 10.1038/s41590-025-02386-2 41540265 PMC12956583

[55] Lewis MJ Barnes MR Blighe K Goldmann K Rana S Hackney JA . Molecular portraits of early rheumatoid arthritis identify clinical and treatment response phenotypes. Cell Rep. (2019) 28:2455–2470.e5. doi: 10.1016/j.celrep.2019.07.091 31461658 PMC6718830

[56] Humby F Lewis M Ramamoorthi N Hackney JA Barnes MR Bombardieri M . Synovial cellular and molecular signatures stratify clinical response to csDMARD therapy and predict radiographic progression in early rheumatoid arthritis patients. Ann Rheum Dis. (2019) 78:761–72. doi: 10.1136/annrheumdis-2018-214539 30878974 PMC6579551

[57] Hashimoto D Chow A Noizat C Teo P Beasley MB Leboeuf M . Tissue-resident macrophages self-maintain locally throughout adult life with minimal contribution from circulating monocytes. Immunity. (2013) 38:792–804. doi: 10.1016/j.immuni.2013.04.004 23601688 PMC3853406

[58] Yona S Kim KW Wolf Y Mildner A Varol D Breker M . Fate mapping reveals origins and dynamics of monocytes and tissue macrophages under homeostasis. Immunity. (2013) 38:79–91. doi: 10.1016/j.immuni.2012.12.001 23273845 PMC3908543

[59] Lee YS Kim MH Yi HS Kim SY Kim HH Kim JH . CX3CR1 differentiates F4/80(low) monocytes into pro-inflammatory F4/80(high) macrophages in the liver. Sci Rep. (2018) 8:15076. doi: 10.1038/s41598-018-33440-9 30305672 PMC6180058

[60] Wan E Yeap XY Dehn S Terry R Novak M Zhang S . Enhanced efferocytosis of apoptotic cardiomyocytes through myeloid-epithelial-reproductive tyrosine kinase links acute inflammation resolution to cardiac repair after infarction. Circ Res. (2013) 113:1004–12. doi: 10.1161/CIRCRESAHA.113.301198 23836795 PMC3840464

[61] Pupjalis D Goetsch J Kottas DJ Gerke V Rescher U . Annexin A1 released from apoptotic cells acts through formyl peptide receptors to dampen inflammatory monocyte activation via JAK/STAT/SOCS signalling. EMBO Mol Med. (2011) 3:102–14. doi: 10.1002/emmm.201000113 21254404 PMC3377061

[62] Komatsu N Takayanagi H . Mechanisms of joint destruction in rheumatoid arthritis - immune cell-fibroblast-bone interactions. Nat Rev Rheumatol. (2022) 18:415–29. doi: 10.1038/s41584-022-00793-5 35705856

[63] Darrieutort-Laffite C Boutet MA Chatelais M Brion R Blanchard F Heymann D . IL-1beta and TNFalpha promote monocyte viability through the induction of GM-CSF expression by rheumatoid arthritis synovial fibroblasts. Mediators Inflammation. (2014) 2014:241840. doi: 10.1155/2014/241840 25484525 PMC4251793

[64] Korsunsky I Wei K Pohin M Kim EY Barone F Major T . Cross-tissue, single-cell stromal atlas identifies shared pathological fibroblast phenotypes in four chronic inflammatory diseases. Med. (2022) 3:481–518.e14. doi: 10.1016/j.medj.2022.05.002 35649411 PMC9271637

[65] Rose-John S . IL-6 trans-signaling via the soluble IL-6 receptor: importance for the pro-inflammatory activities of IL-6. Int J Biol Sci. (2012) 8:1237–47. doi: 10.7150/ijbs.4989 23136552 PMC3491447

[66] Kuo D Ding J Cohn IS Zhang F Wei K Rao DA . HBEGF+ macrophages in rheumatoid arthritis induce fibroblast invasiveness. Sci Transl Med. (2019) 11:eaau8587. doi: 10.1126/scitranslmed.aau8587 31068444 PMC6726376

[67] Tunyogi-Csapo M Kis-Toth K Radacs M Farkas B Jacobs JJ Finnegan A . Cytokine-controlled RANKL and osteoprotegerin expression by human and mouse synovial fibroblasts: fibroblast-mediated pathologic bone resorption. Arthritis Rheum. (2008) 58:2397–408. doi: 10.1002/art.23653 18668542

[68] O'Brien W Fissel BM Maeda Y Yan J Ge X Gravallese EM . RANK-independent osteoclast formation and bone erosion in inflammatory arthritis. Arthritis Rheumatol. (2016) 68:2889–900. doi: 10.1002/art.39837 27563728 PMC5125876

[69] Harre U Georgess D Bang H Bozec A Axmann R Ossipova E . Induction of osteoclastogenesis and bone loss by human autoantibodies against citrullinated vimentin. J Clin Invest. (2012) 122:1791–802. doi: 10.1172/JCI60975 22505457 PMC3336988

[70] Krishnamurthy A Joshua V Haj Hensvold A Jin T Sun M Vivar N . Identification of a novel chemokine-dependent molecular mechanism underlying rheumatoid arthritis-associated autoantibody-mediated bone loss. Ann Rheum Dis. (2016) 75:721–9. doi: 10.1136/annrheumdis-2015-208093 26612338 PMC4819614

[71] Harre U Lang SC Pfeifle R Rombouts Y Fruhbeisser S Amara K . Glycosylation of immunoglobulin G determines osteoclast differentiation and bone loss. Nat Commun. (2015) 6:7651. doi: 10.1038/ncomms7651 25825024 PMC4389255

[72] Diarra D Stolina M Polzer K Zwerina J Ominsky MS Dwyer D . Dickkopf-1 is a master regulator of joint remodeling. Nat Med. (2007) 13:156–63. doi: 10.1038/nm1538 17237793

[73] Jha AK Huang SC Sergushichev A Lampropoulou V Ivanova Y Loginicheva E . Network integration of parallel metabolic and transcriptional data reveals metabolic modules that regulate macrophage polarization. Immunity. (2015) 42:419–30. doi: 10.1016/j.immuni.2015.02.005 25786174

[74] Tannahill GM Curtis AM Adamik J Palsson-McDermott EM McGettrick AF Goel G . Succinate is an inflammatory signal that induces IL-1beta through HIF-1alpha. Nature. (2013) 496:238–42. doi: 10.1038/nature11986 23535595 PMC4031686

[75] Brouwer E Gouw AS Posthumus MD van Leeuwen MA Boerboom AL Bijzet J . Hypoxia inducible factor-1-alpha (HIF-1alpha) is related to both angiogenesis and inflammation in rheumatoid arthritis. Clin Exp Rheumatol. (2009) 27:945–51 20149310

[76] Moreland LW Baumgartner SW Schiff MH Tindall EA Fleischmann RM Weaver AL . Treatment of rheumatoid arthritis with a recombinant human tumor necrosis factor receptor (p75)-Fc fusion protein. N Engl J Med. (1997) 337:141–7. doi: 10.1056/NEJM199707173370301 9219699

[77] Lipsky PE van der Heijde DM St Clair EW Furst DE Breedveld FC Kalden JR . Infliximab and methotrexate in the treatment of rheumatoid arthritis. N Engl J Med. (2000) 343:1594–602. doi: 10.1056/NEJM200011303432202 11096166

[78] Smolen JS Beaulieu A Rubbert-Roth A Ramos-Remus C Rovensky J Alecock E . Effect of interleukin-6 receptor inhibition with tocilizumab in patients with rheumatoid arthritis (OPTION study): a double-blind, placebo-controlled, randomised trial. Lancet. (2008) 371:987–97. doi: 10.1016/S0140-6736(08)60453-5 18358926

[79] Taylor PC Keystone EC van der Heijde D Weinblatt ME Del Carmen Morales L Reyes Gonzaga J . Baricitinib versus placebo or adalimumab in rheumatoid arthritis. N Engl J Med. (2017) 376:652–62. doi: 10.1056/NEJMoa1608345 28199814

[80] van Vollenhoven RF Fleischmann R Cohen S Lee EB Garcia Meijide JA Wagner S . Tofacitinib or adalimumab versus placebo in rheumatoid arthritis. N Engl J Med. (2012) 367:508–19. doi: 10.1056/NEJMoa1112072 22873531

[81] Fleischmann R Pangan AL Song IH Mysler E Bessette L Peterfy C . Upadacitinib versus placebo or adalimumab in patients with rheumatoid arthritis and an inadequate response to methotrexate: results of a phase III, double-blind, randomized controlled trial. Arthritis Rheumatol. (2019) 71:1788–800. doi: 10.1002/art.41032 31287230

[82] Kremer JM Westhovens R Leon M Di Giorgio E Alten R Steinfeld S . Treatment of rheumatoid arthritis by selective inhibition of T-cell activation with fusion protein CTLA4Ig. N Engl J Med. (2003) 349:1907–15. doi: 10.1056/NEJMoa035075 14614165

[83] Bresnihan B Alvaro-Gracia JM Cobby M Doherty M Domljan Z Emery P . Treatment of rheumatoid arthritis with recombinant human interleukin-1 receptor antagonist. Arthritis Rheum. (1998) 41:2196–204. doi: 10.1002/1529-0131(199812)41:12<2196::AID-ART15>3.0.CO;2-2 9870876

[84] Edwards JCW Szczepanski L Szechinski J Filipowicz-Sosnowska A Emery P Close DR . Efficacy of B-cell-targeted therapy with rituximab in patients with rheumatoid arthritis. N Engl J Med. (2004) 350:2572–81. doi: 10.1056/NEJMoa032534 15201414

[85] Xie W Chen T Xiao S Huang H Zhang Z . Difficult-to-treat rheumatoid arthritis: systematic review and meta-analysis on global prevalence. Ann Rheum Dis. (2026) 85:417–24. doi: 10.1016/j.ard.2025.10.007 41188120

[86] Nagy GR Roodenrijs NMT Welsing PMJ Kedves M Hamar A van der Goes MC . EULAR points to consider for the management of difficult-to-treat rheumatoid arthritis. Ann Rheum Dis. (2022) 81:20–33. doi: 10.1136/annrheumdis-2021-220973 34407926 PMC8761998

[87] Roodenrijs NMT Welsing PMJ van Roon J Schoneveld JLM van der Goes MC Nagy G . Mechanisms underlying DMARD inefficacy in difficult-to-treat rheumatoid arthritis: a narrative review with systematic literature search. Rheumatology. (2022) 61:3552–66. doi: 10.1093/rheumatology/keac114 35238332 PMC9434144

[88] Nerviani A Di Cicco M Mahto A Lliso-Ribera G Rivellese F Thorborn G . A pauci-immune synovial pathotype predicts inadequate response to TNFα-blockade in rheumatoid arthritis patients. Front Immunol. (2020) 11:845. doi: 10.3389/fimmu.2020.00845 32431716 PMC7214807

[89] Peres RS Liew FY Talbot J Carregaro V Oliveira RD Almeida SL . Low expression of CD39 on regulatory T cells as a biomarker for resistance to methotrexate therapy in rheumatoid arthritis. Proc Natl Acad Sci USA. (2015) 112:2509–14. doi: 10.1073/pnas.1424792112 25675517 PMC4345589

[90] Degboé Y Rauwel B Baron M Boyer JF Ruyssen-Witrand A Constantin A . Polarization of rheumatoid macrophages by TNF targeting through an IL-10/STAT3 mechanism. Front Immunol. (2019) 10:3. doi: 10.3389/fimmu.2019.00003 30713533 PMC6345709

[91] Nguyen DX Ehrenstein MR . Anti-TNF drives regulatory T cell expansion by paradoxically promoting membrane TNF-TNF-RII binding in rheumatoid arthritis. J Exp Med. (2016) 213:1241–53. doi: 10.1084/jem.20151255 27270893 PMC4925013

[92] Diller M Hasseli R Hülser ML Aykara I Frommer K Rehart S . Targeting activated synovial fibroblasts in rheumatoid arthritis by peficitinib. Front Immunol. (2019) 10:541. doi: 10.3389/fimmu.2019.00541 30984167 PMC6448044

[93] De Lima J Boutet MA Bortolotti O Chépeaux LA Glasson Y Dumé AS . Spatial mapping of rheumatoid arthritis synovial niches reveals a LYVE1+ macrophage network associated with response to therapy. Ann Rheum Dis. (2025) 84:1955–67. doi: 10.1016/j.ard.2025.07.019 40849271

[94] Humby F Durez P Buch MH Lewis MJ Rizvi H Rivellese F . Rituximab versus tocilizumab in anti-TNF inadequate responder patients with rheumatoid arthritis (R4RA): 16-week outcomes of a stratified, biopsy-driven, multicentre, open-label, phase 4 randomised controlled trial. Lancet. (2021) 397:305–17. doi: 10.1016/S0140-6736(20)32341-2 33485455 PMC7829614

[95] Cohen SB Dore RK Lane NE Ory PA Peterfy CG Sharp JT . Denosumab treatment effects on structural damage, bone mineral density, and bone turnover in rheumatoid arthritis: a twelve-month, multicenter, randomized, double-blind, placebo-controlled, phase II clinical trial. Arthritis Rheum. (2008) 58:1299–309. doi: 10.1002/art.23417 18438830

[96] Smolen JS Landewe RBM Bergstra SA Kerschbaumer A Sepriano A Aletaha D . EULAR recommendations for the management of rheumatoid arthritis with synthetic and biological disease-modifying antirheumatic drugs: 2022 update. Ann Rheum Dis. (2023) 82:3–18. doi: 10.1136/ard-2022-223356 36357155

[97] Sepriano A Kerschbaumer A Bergstra SA Smolen JS van der Heijde D Caporali R . Safety of synthetic and biological DMARDs: a systematic literature review informing the 2022 update of the EULAR recommendations for the management of rheumatoid arthritis. Ann Rheum Dis. (2023) 82:107–18. doi: 10.1136/ard-2022-223357 36376026

[98] Norling LV Headland SE Dalli J Arnardottir HH Haworth O Jones HR . Proresolving and cartilage-protective actions of resolvin D1 in inflammatory arthritis. JCI Insight. (2016) 1:e85922. doi: 10.1172/jci.insight.85922 27158677 PMC4855303

[99] Proudman SM James MJ Spargo LD Metcalf RG Sullivan TR Rischmueller M . Fish oil in recent onset rheumatoid arthritis: a randomised, double-blind controlled trial within algorithm-based drug use. Ann Rheum Dis. (2015) 74:89–95. doi: 10.1136/annrheumdis-2013-204145 24081439

[100] Patel HB Kornerup KN Sampaio AL D'Acquisto F Seed MP Girol AP . The impact of endogenous annexin A1 on glucocorticoid control of inflammatory arthritis. Ann Rheum Dis. (2012) 71:1872–80. doi: 10.1136/annrheumdis-2011-201180 22562975 PMC3440300

[101] Burmester GR McInnes IB Kremer J Miranda P Korkosz M Vencovsky J . A randomised phase IIb study of mavrilimumab, a novel GM-CSF receptor alpha monoclonal antibody, in the treatment of rheumatoid arthritis. Ann Rheum Dis. (2017) 76:1020–30. doi: 10.1136/annrheumdis-2016-210624 28213566

[102] Fleischmann RM van der Heijde D Strand V Atsumi T McInnes IB Takeuchi T . Anti-GM-CSF otilimab versus tofacitinib or placebo in patients with active rheumatoid arthritis and an inadequate response to conventional or biologic DMARDs: two phase 3 randomised trials (contRAst 1 and contRAst 2). Ann Rheum Dis. (2023) 82:1516–26. doi: 10.1136/ard-2023-224482 37699654 PMC10646845

[103] Weinblatt ME Taylor PC McInnes IB Atsumi T Strand V Takeuchi T . Long-term safety and efficacy of anti-GM-CSF otilimab in patients with rheumatoid arthritis: long-term extension of three phase 3 randomised trials (contRAst X). BMJ Open. (2025) 15:e088869. doi: 10.1136/bmjopen-2024-088869 40044198 PMC11883618

[104] Tuttle J Drescher E Simon-Campos JA Emery P Greenwald M Kivitz A . A phase 2 trial of peresolimab for adults with rheumatoid arthritis. N Engl J Med. (2023) 388:1853–62. doi: 10.1056/NEJMoa2209856 37195941

[105] Hegarty C Neto N Cahill P Floudas A . Computational approaches in rheumatic diseases - Deciphering complex spatio-temporal cell interactions. Comput Struct Biotechnol J. (2023) 21:4009–20. doi: 10.1016/j.csbj.2023.08.005 37649712 PMC10462794

[106] van den Brink SC Sage F Vértesy Á Spanjaard B Peterson-Maduro J Baron CS . Single-cell sequencing reveals dissociation-induced gene expression in tissue subpopulations. Nat Methods. (2017) 14:935–6. doi: 10.1038/nmeth.4437 28960196

[107] O'Flanagan CH Campbell KR Zhang AW Kabeer F Lim JLP Biele J . Dissociation of solid tumor tissues with cold active protease for single-cell RNA-seq minimizes conserved collagenase-associated stress responses. Genome Biol. (2019) 20:210. doi: 10.1186/s13059-019-1830-0 31623682 PMC6796327

[108] Denisenko E Guo BB Jones M Hou R de Kock L Lassmann T . Systematic assessment of tissue dissociation and storage biases in single-cell and single-nucleus RNA-seq workflows. Genome Biol. (2020) 21:130. doi: 10.1186/s13059-020-02048-6 32487174 PMC7265231

[109] Fritz D Inamo J Zhang F . Single-cell computational machine learning approaches to immune-mediated inflammatory disease: New tools uncover novel fibroblast and macrophage interactions driving pathogenesis. Front Immunol. (2022) 13:1076700. doi: 10.3389/fimmu.2022.1076700 36685542 PMC9846263

[110] Schonfeldova B Zec K Udalova IA . Synovial single-cell heterogeneity, zonation and interactions: a patchwork of effectors in arthritis. Rheumatol (Oxford). (2022) 61:913–25. doi: 10.1093/rheumatology/keab721 34559213 PMC8889290

[111] Kemble S Croft AP . Critical role of synovial tissue-resident macrophage and fibroblast subsets in the persistence of joint inflammation. Front Immunol. (2021) 12:715894. doi: 10.3389/fimmu.2021.715894 34539648 PMC8446662

[112] Hanlon MM Smith CM Canavan M Neto NGB Song Q Lewis MJ . Loss of synovial tissue macrophage homeostasis precedes rheumatoid arthritis clinical onset. Sci Adv. (2024) 10:eadj1252. doi: 10.1126/sciadv.adj1252 39321281 PMC11423874

[113] Hu X Zhang Z Long L Gu M Chen W Pan B . Deconvolution of synovial myeloid cell subsets across pathotypes and role of COL3A1+ macrophages in rheumatoid arthritis remission. Front Immunol. (2024) 15:1307748. doi: 10.3389/fimmu.2024.1307748 38601143 PMC11005452

[114] Gkiouras K Grammatikopoulou MG Myrogiannis I Papamitsou T Rigopoulou EI Sakkas LI . Efficacy of n-3 fatty acid supplementation on rheumatoid arthritis' disease activity indicators: a systematic review and meta-analysis of randomized placebo-controlled trials. Crit Rev Food Sci Nutr. (2024) 64:16–30. doi: 10.1080/10408398.2022.2104210 35900212

[115] Wang W Xu Y Zhou J Zang Y . Effects of omega-3 supplementation on lipid metabolism, inflammation, and disease activity in rheumatoid arthritis: a meta-analysis of randomized controlled trials. Clin Rheumatol. (2024) 43:2479–88. doi: 10.1007/s10067-024-07040-0 38922552

[116] O'Donnell VB Schebb NH Milne GL Murphy MP Thomas CP Steinhilber D . Failure to apply standard limit-of-detection or limit-of-quantitation criteria to specialized pro-resolving mediator analysis incorrectly characterizes their presence in biological samples. Nat Commun. (2023) 14:7172. doi: 10.1038/s41467-023-41766-w 37945602 PMC10636151

[117] Gomez EA Colas RA Souza PR Hands R Lewis MJ Bessant C . Blood pro-resolving mediators are linked with synovial pathology and are predictive of DMARD responsiveness in rheumatoid arthritis. Nat Commun. (2020) 11:5420. doi: 10.1038/s41467-020-19176-z 33110080 PMC7591509

[118] Dalli J Gomez EA . Reply to: Failure to apply standard limit-of-detection or limit-of-quantitation criteria to specialized pro-resolving mediator analysis incorrectly characterizes their presence in biological samples. Nat Commun. (2023) 14:7293. doi: 10.1038/s41467-023-41767-9 37949899 PMC10638266

[119] Kutzner L Rund KM Ostermann AI Hartung NM Galano JM Balas L . Development of an optimized LC-MS method for the detection of specialized pro-resolving mediators in biological samples. Front Pharmacol. (2019) 10:169. doi: 10.3389/fphar.2019.00169 30899221 PMC6416208

[120] Skarke C Alamuddin N Lawson JA Li X Ferguson JF Reilly MP . Bioactive products formed in humans from fish oils. J Lipid Res. (2015) 56:1808–20. doi: 10.1194/jlr.M060392 26180051 PMC4548785

[121] Chidomere CI Wahid M Kemble S Chadwick C Thomas R Hardy RS . Bench to bedside: Modelling inflammatory arthritis. Discov Immunol. (2023) 2:kyac010. doi: 10.1093/discim/kyac010 38567064 PMC10917191

[122] Benson RA McInnes IB Garside P Brewer JM . Model answers: Rational application of murine models in arthritis research. Eur J Immunol. (2018) 48:32–8. doi: 10.1002/eji.201746938 29193037 PMC5814907

[123] Xie W Chen T Chen Y Zhang Z Xiao S . Associated lifestyle factors and comorbidities of difficult-to-treat rheumatoid arthritis: a systematic review and meta-analysis. Lancet Rheumatol. (2026) 8:e425–37. doi: 10.1016/S2665-9913(26)00041-X 41999719

[124] Luciano N Barone E Brunetta E D'Isanto A De Santis M Ceribelli A . Obesity and fibromyalgia are associated with Difficult-to-Treat Rheumatoid Arthritis (D2T-RA) independent of age and gender. Arthritis Res Ther. (2025) 27:2. doi: 10.1186/s13075-024-03432-4 39754234 PMC11697877

[125] David P Di Matteo A Hen O Dass S Marzo-Ortega H Wakefield RJ . Poly-refractory rheumatoid arthritis: An uncommon subset of difficult to treat disease with distinct inflammatory and noninflammatory phenotypes. Arthritis Rheumatol. (2024) 76:510–21. doi: 10.1002/art.42767 38059326

[126] Qi W Robert A Singbo N Ratelle L Fortin PR Bessette L . Characteristics of patients with difficult-to-treat rheumatoid arthritis: a descriptive retrospective cohort study. Adv Rheumatol. (2024) 64:55. doi: 10.1186/s42358-024-00396-6 39107865 PMC13488725

[127] Sun YP Oh SF Uddin J Yang R Gotlinger K Campbell E . Resolvin D1 and its aspirin-triggered 17R epimer. Stereochemical assignments, anti-inflammatory properties, and enzymatic inactivation. J Biol Chem. (2007) 282:9323–34. doi: 10.1074/jbc.M609212200 17244615

[128] Orr SK Colas RA Dalli J Chiang N Serhan CN . Proresolving actions of a new resolvin D1 analog mimetic qualifies as an immunoresolvent. Am J Physiol Lung Cell Mol Physiol. (2015) 308:L904–11. doi: 10.1152/ajplung.00370.2014 25770181 PMC4421783

[129] Rivellese F Nerviani A Giorli G Warren L Jaworska E Bombardieri M . Stratification of biological therapies by pathobiology in biologic-naive patients with rheumatoid arthritis (STRAP and STRAP-EU): two parallel, open-label, biopsy-driven, randomised trials. Lancet Rheumatol. (2023) 5:e648–59. doi: 10.1016/S2665-9913(23)00241-2 38251532

[130] Kraus VB . Biomarkers as drug development tools: discovery, validation, qualification and use. Nat Rev Rheumatol. (2018) 14:354–62. doi: 10.1038/s41584-018-0005-9 29760435

[131] Lin CMA Cooles FAH Isaacs JD . Precision medicine: the precision gap in rheumatic disease. Nat Rev Rheumatol. (2022) 18:725–33. doi: 10.1038/s41584-022-00845-w 36216923

[132] Ciancio A Amati A Gandolfo S Bouyer RG Thomas R Liew DFL . Aligning approaches to rheumatoid arthritis drug development: a comparison of FDA and EMA guidance. Lancet Rheumatol. (2026) 8:e563–75. doi: 10.1016/S2665-9913(26)00086-X 42331475

[133] Brown AK Quinn MA Karim Z Conaghan PG Peterfy CG Hensor E . Presence of significant synovitis in rheumatoid arthritis patients with disease-modifying antirheumatic drug-induced clinical remission: evidence from an imaging study may explain structural progression. Arthritis Rheum. (2006) 54:3761–73. doi: 10.1002/art.22190 17133543

[134] Felson DT Lacaille D LaValley MP Aletaha D . Reexamining remission definitions in rheumatoid arthritis: Considering disease activity score in 28 joints, C-reactive protein, and patient global assessment. ACR Open Rheumatol. (2022) 4:123–7. doi: 10.1002/acr2.11345 34783200 PMC8843760

[135] Genovese MC Cohen S Moreland L Lium D Robbins S Newmark R . Combination therapy with etanercept and anakinra in the treatment of patients with rheumatoid arthritis who have been treated unsuccessfully with methotrexate. Arthritis Rheum. (2004) 50:1412–9. doi: 10.1002/art.20221 15146410

[136] Weinblatt M Combe B Covucci A Aranda R Becker JC Keystone E . Safety of the selective costimulation modulator abatacept in rheumatoid arthritis patients receiving background biologic and nonbiologic disease-modifying antirheumatic drugs: A one-year randomized, placebo-controlled study. Arthritis Rheum. (2006) 54:2807–16. doi: 10.1002/art.22070 16947384

